# Multivalent 2D- and 3D-nanogels as carbohydrate-lectin binders†

**DOI:** 10.1039/d5bm00286a

**Published:** 2025-07-07

**Authors:** Ann-Cathrin Schmitt, Maximilian Braun, Stefanie Wedepohl, Mathias Dimde, Philip Nickl, Kai Ludwig, Tatyana L. Povolotsky, Rainer Haag

**Affiliations:** a Institute of Chemistry and Biochemistry, Freie Universität Berlin Takustr. 3 14195 Berlin Germany haag@chemie.fu-berlin.de; b SupraFAB, Freie Universität Berlin Altensteinstr. 23a 14195 Berlin Germany; c Forschungszentrum für Elektronenmikroskopie (FZEM), Freie Universität Berlin Fabeckstr. 36a 14195 Berlin Germany

## Abstract

The development of synthetic glycoarchitectures for targeted bacterial adhesion represents a promising strategy in anti-adhesion therapy. This study presents the synthesis and characterization of two distinct mannosylated nanogel architectures. First, a spherical 3D-nanogel was prepared *via* nanoprecipitation and functionalized with α-d-mannose units. This system demonstrated enhanced precipitation kinetics in turbidity measurements with Concanavalin A and exhibited single-site binding behavior comparable to monovalent reference compounds when tested with intact *E. coli* strain ORN 178 (FimH^+^) *via* microscale thermophoresis. Cryo-TEM imaging revealed clear co-localization with bacterial pili, confirming specific bacterial interactions. The complementary sheet-like 2D-nanogel, synthesized using a removable graphene template and functionalized with α-d-mannose units, showed distinct dual binding characteristics with significantly different affinities in FimH binding studies. Notably, the high-affinity site of the 2D-nanogel maintained superior binding compared to the 3D architecture. Both architectures were extensively characterized using multiple analytical techniques, confirming their defined structures, sizes, and surface modifications. These findings provide fundamental insights into the influence of spatial ligand presentation on multivalent binding interactions, contributing to the rational design of glycoarchitectures for bacterial targeting.

## Abstract 2

The development of synthetic glycoarchitectures for targeted bacterial adhesion represents a promising strategy in anti-adhesion therapy.

## Graphical abstract

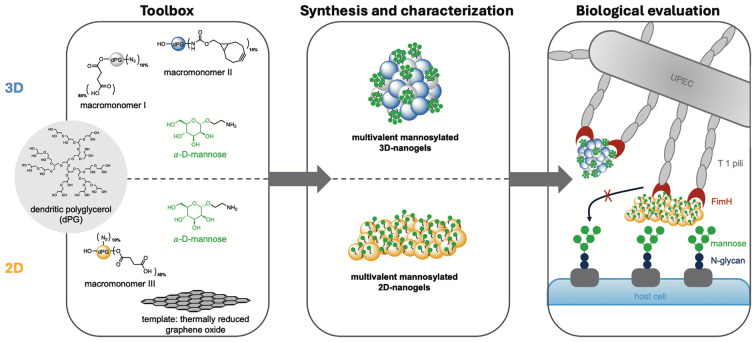

## Introduction

Carbohydrate–protein interactions are fundamental to a wide range of biological processes, including cell recognition, signaling, and immune responses.^1,2^ One key aspect of these interactions is multivalency, which often does not arise from multiple binding sites on a single protein, but rather through protein oligomerization or ligand clustering, where single binding sites create enhanced avidity through increased local rebinding probability. This organization enhances both the strength and specificity of the interaction, making it more effective than monovalent binding events.^3,4^ Overall, the study of carbohydrate–protein interactions (*e.g.* carbohydrate-lectin binding), especially with a focus on multivalency, is essential for understanding many biological mechanisms and can provide insights for developing new therapeutic strategies.

The synthesis of polyglycosylated structures has been significantly developed over the past few decades, resulting in various multivalent nano-sized structures such as polymers,^5^ nanomaterials,^6–8^ and supramolecular structures,^9^ each modified with several carbohydrate units and serving as synthetic mimetics of natural glycoconjugates. Furthermore, previous studies have demonstrated that physicochemical properties of nanomaterials, including lateral dimensions, morphology, surface charge, and functional groups, play a critical role in determining their biological behaviors.^10^ Therefore, the development of multivalent functionalized nanomaterials that mimic the glycocalyx and are defined in their physicochemical properties is of great interest.

Herein, multivalent mannosylated nanogels (Man-NGs) based on crosslinked dendritic polyglycerol (dPG) are described as an attractive class of glycocalyx mimetics. They can be synthesized with precisely controlled sizes and dimensions, and show great potential as a versatile platform for glycosylation. Polymeric NGs have been successfully used in various applications as drug and gene delivery systems,^11–13^ some recent studies have emphasized the potential to either interact with cellular receptors or to function as mimics of such receptors.^14,15^

NGs are particles at the nanometer scale, featuring a tunable size and an internal crosslinked network. They are known for their soft and flexible characteristics, straightforward preparation, and excellent stability.^16,17^ As polymer base for NGs with such application, dPG is an excellent candidate. It is a hyperbranched polymer which is tunable in its molecular weight and size, highly biocompatible, water-soluble and shows low non-specific interactions with biointerfaces.^18^ In addition, the free hydroxyl groups allow the introduction of reactive groups, surface charges or chemical modifications.^19^ To increase the surface area for multivalent display, NGs can be synthesized *via* different synthetic strategies, including the dispersion of preformed polymers *via* salting out, solvent evaporation or by different polymerization techniques (*e.g.* micro- and miniemulsion or nanoprecipitation).^20,21^ Many of these techniques, however, depend on the use of surfactants, which can pose challenges for biomedical applications if not thoroughly purified. To address this issue, surfactant-free methods for synthesizing nanogels have been developed, utilizing techniques such as Cu(i)-catalyzed azide–alkyne cycloaddition (CuAAC),^22^ thiol-Michael addition,^11^ strain-promoted azide–alkyne cycloaddition (SPAAC),^23,24^ and inverse electron demand Diels–Alder (iEDDA) cyclization through inverse nanoprecipitation under mild conditions.^25^ These techniques lead to the formation of three-dimensional (3D) spherical NGs, which possess a well-defined architecture and uniform size, but a low ability to adapt their shape to small organisms such as viruses or bacteria. Therefore, they can only provide a curvature-dependent contact area at the interfaces between the organism and the nanomaterial.^7^ In order to increase the flexibility of NGs, a template-assisted synthesis of two-dimensional (2D)-NGs based on dPG was considered. In the past years it was shown that the attachment of dPG on the surface of templates resulted in 2D platforms with a high potential in biomedical application.^6,26^ The toxicity associated with the graphene template to which dPG is attached however poses a significant challenge, undermining the benefits of these systems.^27^ Therefore, our group developed a method in which dPG is covalently bound to the surface of the flexible graphene template *via* a pH-sensitive linker. The covalent binding allows the dPG units to be clicked together using CuAAC, resulting in 2D-NGs.^27^ By lowering the pH value, it is possible to completely remove the graphene and at the same time the resulting 2D-NGs are sufficiently stable for subsequent functionalization.

In this work we present multivalent 2D- and 3D-NGs based on dPG by using a flexible graphene sheet template and inverse nanoprecipitation, respectively. We sought to synthesize NGs in a size range of 200–250 nm with small size distributions and a strong negative zeta potential. Afterwards, 2D- and 3D-NGs were mannosylated on the surface *via* active ester functionalization, resulting in what we term Man-NGs. α-d-Mannose was selected for the glycosylation of NGs due to its specific binding affinity for Concanavalin A (ConA) and FimH-expressing *Escherichia coli* (*E. coli*). FimH is an adhesin located at the tip of the piliae of certain bacteria, allowing them to adhere to host cells by recognizing and binding to mannose residues.^28^ This adhesion mechanism enables pathogenic bacteria, particularly uropathogenic *E. coli* (UPEC), to cause severe urinary tract infections (UTIs), which can progress to pyelonephritis, urosepsis, and in some cases systemic infections leading to septicemia. The interactions of the Man-NGs with the desired lectin ConA were investigated by an precipitation assay. In a second step, binding of Man-NGs to FimH expressing *E. coli* was investigated by microscale thermophoresis (MST) and cryogenic transmission electron microscopy (cryo-TEM) experiments.

## Experimental

### Materials

Anhydrous solvents were chemically dried by conventional methods prior to use or commercially purchased from Acros Organics in septum-sealed bottles. All reactions which involved air- or water-sensitive compounds were carried out in a dried flask under an argon atmosphere. Water was used from a Milli-Q station from Millipore (Merck, Millipore). Dendritic polyglycerol (dPG) with a molecular weight of 10 kDa, dPG-(N_3_)_10%_ and dPG-(NH_2_)_10%_ were synthesized according to an established procedure. Since dPG is a hygroscopic polymer, it was dried (50 °C, 10–3 mbar, 24 h) prior to reactions. Methane sulfonyl chloride (MsCl), triethylamine (TEA), cyanuric chloride, sodium azide, tris(2-chlorehyl) phosphate (TCEP), succinic anhydride, 3-azido propanol, diisopropylethylamine (DIPEA), bicyclo[6.1.0] non-4-yn-9-ylmethyl-(4-nitrophenyl) carbonate (BCN), *N*,*N*,*N*′,*N*′-tetramethyl-*O*-(*N*-succinimidyl) uronium hexafluorophosphate (HSTU), *N*-methyl-2-pyrrolidone (NMP), *p*-toluene sulfonic acid (PTSA), methyl α-d-mannopyranoside (MeMan), tripropargylamine, sodium ascorbate (NaAsc), copper(ii) sulfate pentahydrate (CuSO_4_·5H_2_O), palladium on carbon 10 wt%, and benzoylated dialysis tubes with a molecular weight cut-off (MWCO) of 2 kDa were purchased from Sigma-Aldrich. Spectra/POR dialysis membrane (50 kDa MWCO) were purchased from Carl Roth. Moreover, the following compounds were acquired: 2′-azidoethyl α-d-mannopyranoside (Biosynth Ltd), thermally reduced graphene oxide (TRGO) was kindly provided by Prof. Rolf Mülhaupt,^29^ 3-(4-(dimethoxymethyl)phenoxy) propan-1-amine (DMPA) was synthesized and kindly provided by Sebastian Schötz.^25^

^1^H and ^13^C NMR spectra were recorded on a Jeol Eclipse 500 MHz (Tokyo, Japan) instrument. Deuterated solvents used are indicated in each case. Chemical shifts *δ* are expressed in parts per million (ppm) relative to tetramethylsilane (TMS) as an internal standard or relative to the resonance of the solvent (^1^H NMR: CDCl_3_: *δ* = 7.26 ppm, DMSO-d_6_: *δ* = 2.50 ppm; D_2_O: *δ* = 4.79 ppm; ^13^C NMR: CDCl_3_: *δ* = 77.00 ppm, DMSO-d_6_: *δ* = 39.70 ppm). All spectra were recorded at room temperature.

### Synthetic procedures

#### Synthesis of dendritic polyglycerol (dPG)-based macromolecules

dPG 1, dPG-(N_3_)_10%_2, dPG-(NH_2_)_10%_3, and dPG-(BCN)_10%_4 were synthesized according to established protocols.^19,30–32^ Briefly, the hydroxyl groups of dPG 1 (*M*_n_ = 10 kDa) were first mesylated and then converted to azides using sodium azide, resulting in dPG-(N_3_)_10%_2. Afterwards dPG-(N_3_)_10%_2 was reduced to dPG-(NH_2_)_10%_3 using TCEP and subsequently coupled BCN to achieve the product dPG-(BCN)_10%_4.

#### dPG-(N_3_)_10%_–(COOH)_*x*%_ (*x* = 40 or 80) 5, 6

dPG-(N_3_)_10%_2 (1.0 g, 0.1 mmol, 1.0 eq.) was dissolved in 5 mL anhydrous DMF. Succinic anhydride (1.2 g, 11.7 mmol, 1.2 eq. per OH-group) was added and the mixture was stirred for 24 h at 60 °C. The resulting solution was dialyzed for 2 d against MeOH (MWCO 2 kDa). The solvent was removed in vacuum and dPG-(N_3_)_10%_–(COOH)_80%_5 was received (1.2 g, 0.1 mmol, 80% conversion, 85%). Analogously, dPG-(N_3_)_10%_–(COOH)_40%_6 was synthesized, aiming for 40% conversion of hydroxyls to carboxylic acids.

^1^H NMR (500 MHz, D_2_O, *δ* (ppm)): 4.50–3.30 (m, 5H, polyglycerol backbone (H1–H3)), 2.85–2.25 (m, 4H, H4, H5).

#### Preparation of 3D-nanogels (3D-NGs)

dPG-(N_3_)_10%_–(COOH)_80%_5 (15.0 mg, 25.5 μmol N_3_-groups) and dPG-(BCN)_10%_4 (10.0 mg, 27.4 μmol alkyne groups) were dissolved separately in 1 mL MeOH, respectively. The solutions were mixed, vortexed and injected into acetone (750 mL) under vigorous stirring (1500 rpm). The reaction was quenched after 25 min by addition of 3-azido propanol (50 μg). After 24 h, Milli-Q water (100 mL) was added, the non-solvent was removed under reduced pressure. The resulting 3D-NG 7 was dialyzed against Milli-Q water for 72 h using a MWCO 50 kDa membrane and stored in solution.

#### 3D-nanogel characterization

The size and the zeta potential (ZP) measurements of the NGs were recorded by dynamic light scattering (DLS) performed on a Malvern Zetasizer Ultra/Pro (Malvern Instruments Ltd) using an He–Ne laser (*λ* = 532 nm) at 173° backscatter and automated attenuation at 25 °C, unless stated otherwise. All sample measurements were performed in triplicate, yielding a mean size value plus standard deviation. Further, the diameter of the NGs was characterized by cryogenic transmission electron microscopy (cryo-TEM).

#### Synthesis of a 2D-graphene template

G-Trz 9 and G-Linker 10 were synthesized according to previous reported procedures.^33,34^

#### G-dPG 11

G-Linker 10 (50.0 mg) was dispersed in dry DMF (80 mL) and stirred overnight. The mixture was sonicated for 20 min. Afterwards, dPG-(N_3_)_10%_–(COOH)_40%_6 (450.0 mg, 0.04 mmol) was dissolved in dry DMF (10 mL) and PTSA (67.0 mg, 0.4 mmol) was added to the mixture and stirred for 24 h at room temperature. Vacuum was applied every 30 min to remove the produced MeOH. The black product G-dPG 11 (460.4 mg) was obtained by lyophilizing after 2 d dialysis (MWCO 50 kDa) against H_2_O : MeOH (1 : 2).

#### Synthesis of 2D-nanogels (2D-NGs)

G-dPG 11 (340 mg) was suspended in DMF : H_2_O (30 mL, 1 : 1), sonicated for 30 min, and bubbled with argon for 15 min. Tripropargylamine (0.2 mL) was added. 100 μL each of NaAsc (*c* = 90 mg mL^−1^) and CuSO_4_·5H_2_O (*c* = 60 mg mL^−1^) were then added dropwise to the solution and stirred at 50 °C overnight. Every 12 h, the mixture was sonicated for 15 min and the same amount of NaAsc and CuSO_4_·5H_2_O were added. After 48 h, the pH-value was adjusted to a value of 3 with 1 M HCl and stirred for 24 h. Then, the mixture was centrifugated two times (10 000 rpm, 45 min) and the residue was washed with H_2_O in between. The product 2D-NG 12 was dialyzed against H_2_O for 48 h (MWCO 50 kDa) and stored in the fridge at 4 °C.

#### General synthesis procedure for Man-NGs 8, 13

(2-Aminoethyl)-α-d-mannopyranoside (Man-NH_2_) 14 was synthesized according to a published procedure.^35^ First, NGs 7 and 12 were transferred into DMF with subsequent evaporation of H_2_O using a vacuum line. All carboxylic groups in the NGs were converted with 1.5 eq. of DIPEA as well as HSTU into *N*-hydroxysuccinimid (NHS) esters for amide coupling. Active esters were formed *in situ* under argon atmosphere and stirred overnight at r.t. Afterwards, 1.0 eq. per carboxylic group of Man-NH_2_14 was added and the reaction was stirred at room temperature for 24 h. The products 3D-Man 8 and 2D-Man 13 were each dialyzed (MWCO 50 kDa) against Milli-Q water for 48 h and stored in solution at 4 °C. The amount of α-d-mannose on the surface of Man-NGs was determined following the H_2_SO_4_/UV-Vis assay described by Albalasmeh *et al.*^36^

### Methods

#### Transmission electron microscopy (TEM)

For TEM measurements, droplets of the sample solution (5 μL) were applied on hydrophilized Formvar®-supported carbon-coated copper grids (400 mesh) for 60 s. Hydrophilization was achieved beforehand by 60 s glow discharging in a Emscope SC 500 device at 20 mA. The supernatant fluid was removed by blotting with a filter paper and the sample was allowed to dry in air. A standard holder was used to transfer the dried samples into a TALOS L120C electron microscope (Thermo Fisher Scientific Inc.) The microscope was equipped with a Tungsten filament and operated at 120 kV acceleration voltage. Micrographs were acquired on a FEI Ceta CMOS camera (Thermo Fisher Scientific Inc.) at a nominal magnification of 36 000×, corresponding to a calibrated pixel size of 4.09 Å per pixel.

#### Cryogenic transmission electron microscopy (cryo-TEM)

The samples were vitrified by plunge freezing in liquid ethane using an automated vitrification robot (FEI Vitrobot Mark III, Fisher Scientific). For the preparation, a droplet (4 μL) of the sample solution was placed on hydrophilized holey carbon filmed grids (Quantifoil R1/4 from Quantifoil Micro Tools GmbH, Großlöbichau, Germany) After automatic blotting and plunge freezing in liquid ethane the grids were stored in liquid nitrogen prior to the measurement.

Cryo-TEM measurements were performed on a Talos Arctica transmission electron microscope (Thermo Fisher Scientific Inc.). The vitrified grids were stabilized by a copper autogrid and fixed with a spring clamp under liquid nitrogen. These autogrids were transferred under liquid nitrogen into the transmission electron microscope using the microscope's autoloader transfer routine. Micrographs were recorded using the microscopes low-dose protocol at a primary magnification of 28 000× and an acceleration voltage of 200 kV. Images were recorded by a Falcon 3CE direct electron detector (48 aligned frames) at full size (4 k). The defocus was chosen to be 5 μm in all cases to create sufficient phase contrast.

#### Pseudo cryogenic electron tomography (pseudo cryo-ET)

Dried grids were clipped and transferred into the Talos Arctica transmission electron microscope under liquid nitrogen. Tomographic tilt series were recorded using the FEI Tomography software (Version 4.5.0, ThermoFisher Scientific Inc.) in the tilt range of ±64° at 2° increments at a primary magnification of 28 k. Tilt-images were recorded with the Falcon 3CE direct electron detector at full image size (4.096 Å per pixel) and an exposure time of 1.22 s per image. The defocus was chosen to be −2 to 5 μm to create sufficient phase contrast. Reconstruction of the tomograms was carried out with binned data (binning factor 2) using the ThermoFisher Inspect 3D software, version 4.5.2. The 3D voltex presentation was prepared using ThermoFisher AMIRA 3D.

#### X-ray photoelectron spectroscopy (XPS)

XPS spectra were recorded on a Kratos (Manchester, UK) Axis Ultra DLD spectrometer, equipped with a monochromatic Al Kα X-ray source. The spectra were measured in normal emission, and a source-to-sample angle of 60° was used. All spectra were recorded utilizing the fixed analyzer transmission (FAT) mode. The binding energy scale of the instrument was calibrated, following a technical procedure provided by Kratos Analytical Ltd (calibration was performed according to ISO 15472). The spectra were recorded utilizing the instrument's slot and hybrid lens modes. An analysis area of approximately 300 μm × 700 μm was investigated; charge neutralization was applied. For quantification, the survey spectra were measured with a pass energy of 80 eV, and the spectra were quantified utilizing the empirical sensitivity factors that were provided by Kratos. The high-resolution XP spectra were measured with a pass energy of 20 eV, and the respective data were processed using UNIFIT spectrum processing software. For fitting, a Shirley background and a Gaussian/Lorentzian sum function and peak shape model GL (30) were used. All binding energies were calibrated to the signal observed for the aliphatic C–C bond component (observed at binding energy of 284.8 eV).

#### H_2_SO_4_/UV-Vis assay

UV-Vis spectroscopy was used to determine the DF of α-d-mannose on the surface of the Man-NGs. UV-Vis absorbance measurements were performed on an Agilent Cary 8654 UV-Vis spectrophotometer at 20 °C with a Julabo 200F temperature controller using half-micro plastic cuvettes. The assay was performed following a procedure described by Albalasmeh *et al.*^36^ The carbohydrate content could be quantified using UV-Vis spectroscopy at 315 nm in an H_2_SO_4_/H_2_O solution, following the creation of a calibration curve based on methyl α-d-mannopyranoside (MeMan). The weight percentage of mannose was calculated by the following equation, where *W*_mannose_ and *W*_NG_ represent the mannose weight and the weight of the NGs, respectively.Man-NGs loading (wt%) = (*W*_mannose_/*W*_NG_) × 100

#### ConA precipitation assay

ConA (Sigma # L7647) was solubilized in 0.1 M Tris, 80 mM glycine pH 5.0, 3 mM CaCl_2_ and 3 mM MnCl_2_ at a concentration of 3 mg mL^−1^. For the assay, this stock solution was diluted in PBS (with Ca and Mg) to 0.3 mg mL^−1^ and 100 μL per well was filled into one half of a U-bottom transparent 96 well plate. The NGs and yeast mannan (YMan) (Sigma # M7504) were 2-fold serially diluted in the wells of the other half of the plate, starting with the highest concentration of 0.2 mg mL^−1^ in 100 μL per well. Absorbance was read at 340 nm in a microplate reader (Tecan SPARK). Then, 100 μL per well ConA were transferred from one side of the plate to the 100 μL per well of compound dilutions, shaken (orbital) for 5 s with an amplitude of 1 mm and 510 rpm and absorbance was read every 30 s for 20 min.

Three independent plates were prepared the same way and combined to calculate mean and standard deviation (SD) of the kinetic curves. To explore other ratios, variants of this assay were performed with different dilutions and volumes of compounds or at constant compound concentration and varying ConA concentrations. The plates were then allowed to stand at r.t. for 1 h and afterwards centrifuged for 15 min at 1000 rpm. 5 μL of the supernatant was removed and pipetted to another well plate for protein assay. A standard dilution series of ConA was prepared in this plate. 200 μL per well of Bradford reagent was added to standards and samples, and after 5 min shaking at r.t., absorbance was read at 595 nm.

#### Microscale thermophoresis (MST)

Binding affinities were determined *via* MST using the Monolith NT.115Pico instrument with premium capillaries (SKU: MO-K025), both from Nanotemper. The binding affinities of intact *E. coli* strain ORN 178 and the following compounds were measured: 3D-Man, 2D-Man, MeMan (positive control), 3D-NG and 2D-NG (both negative controls). *E. coli* ORN 178 was inactivated using ROTI® Histofix (Carl Roth) and labeled with propidium iodide (PI). The protocol is an adapted version from Hanheiser *et al.*^37^

For the MST measurement, the bacteria solution was prepared as follows: *E. coli* strain ORN178 was freshly streaked out from frozen stock and single-colony was inoculated into LB-medium (5.00 mL) and allowed to grow to mid-exponential phase (OD_600_ 0.6) at 37 °C at 250 rpm. The bacteria were washed with DPBS and resuspended in 5 mL of Histofix. After an incubation time of 30 min at room temperature using a rocking shaker at moderate speed. Subsequently, the culture was centrifuged at 3000 rpm for 5 min and the supernatant was discarded. The bacterial pellet was resuspended in DPBS before being centrifuged again. The supernatant was again discarded, and the pellet was resuspended in 2 mL in DPBS. The OD_600_ of the resulting culture was measured. 2 μL of PI was added and allowed to react at room temperature for 30 min in dark conditions. The bacteria were diluted to a final concentration of OD = 1.0, where this was calculated to be equivalent to a concentration on 10 nM, before use in MST measurement and stored on ice and in dark conditions. The tested compounds were serially 2-fold diluted in DPBS with 0.05% (v/v) Tween (PBST) with a resulting volume of 6 μL. 6 μL of *E. coli* ORN178 at a concentration of 10 nM was added to the mixture resulting in a final volume of 12 μL. The final mixture of PBST, tested compound and labeled *E. coli* ORN178 was vortexed, spun down and equilibrated at room temperature for 60 seconds before collecting the mixtures in premium capillaries for measurement. MST measurements were performed at 22 °C over a 30 s time interval using the MO. Control software (Nanotemper) at 80% Red-LED excitation and 40% IR-laser power. The resultant data were analyzed *via* Affinity Analysis Software v2.3 (Nanotemper), exported and plotted with Prism 10.0.2 (Graphpad by Dotmatics). Four biological repeats (*N*) were performed per bacteria-compound combination. *K*_D_ values were calculated using the *K*_D_ model and a signal to noise ratio of 5.0, as well as a response amplitude of 12 was required to establish binding. For 3D-Man samples two separate 2-fold serial dilutions were carried out the first was from a stock solution of 5 mM (16 2-fold serial dilutions) and the second from a starting stock solution of 0.5 mM (16 2-fold serial dilutions). The resulting data was then combined for analysis. The two highest concentrations of 2.5 mM and 1.25 mM were excluded from the final analysis due to extreme aggregation of the sample.

## Results and discussion

### Synthesis and characterization of nanogel precursors

**Fig. 1:**
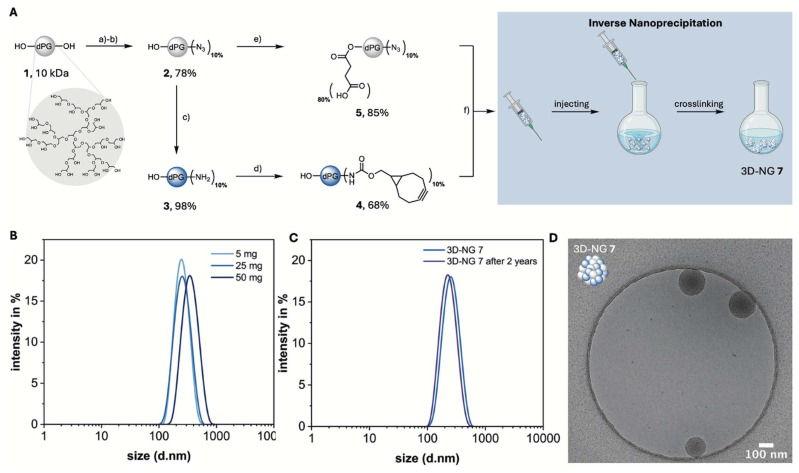
Synthesis and characterization of 3D-NGs. (A) Synthesis of macromolecular precursors followed by NG synthesis *via* inverse nanoprecipitation. Reagents and conditions: (a) MsCl, TEA, DMF, 0 °C to 80 °C, 48 h; (b) NaN_3_, DMF, 60 °C, 72 h; (c) TCEP, MeOH, H_2_O, r.t., 24 h (d) BCN, TEA, DMF, r.t., 24 h; (e) succinic anhydride, DMF, 60 °C, 24 h; (f) MeOH, 3-azidopropanol, r.t., 24 h. (B) Particle size by DLS of different batches of 3D-NG (1 mg mL^−1^ in PB (pH = 7.4) at 25 °C). (C) Stability measurement of 3D-NG 7 after two years under same conditions. (D) Cryo-TEM image of 3D-NG 7 at the concentration of 2 mg mL^−1^ in Mili-Q water, showing spherical structures.

In the present study, mannosylated nanogels (Man-NGs) with varying architectural features were designed and synthesized to investigate their bacterial-binding properties. These NGs were prepared *via* strain-promoted azide–alkyne cycloaddition (SPAAC) using macromolecular dPG-based precursors as the structural backbone (Fig. 1A). First, 10% of the hydroxyl groups of a 10 kDa dPG 1 were converted in two reaction steps into azide groups according to a well-established procedure to obtain dPG-(N_3_)_10%_2 in an overall yield of 92%.^19^ The structure of dPG-(N_3_)_10%_2 was confirmed by ^1^H NMR and FTIR (Fig. S1–S3†). To achieve the first macromolecular precursor dPG-(BCN)_10%_4, dPG-(N_3_)_10%_2 was subsequently reduced in a Staudinger reaction with TCEP to dPG-(NH_2_)_10%_3.^38^ The reaction was monitored by FTIR and proceeded quantitative (Fig. S4†). Then, by using the activated carbonate of BCN, dPG-(NH_2_)_10%_3 was decorated with the necessary alkynes to the first macromolecule dPG-(BCN)_10%_4 in a yield of 89%.^31^ The structure was confirmed by ^1^H NMR (Fig. S5†).

For the second macromolecular precursor dPG-(N_3_)_10%_–(COOH)_80%_5, 80% of the hydroxyl groups of dPG-(N_3_)_10%_2 were converted with succinic anhydride into carboxylic acid groups which enables post-functionalization of the NGs. The success of the reaction was confirmed by ^1^H NMR (Fig. S6†). The same procedure was applied to produce another precursor, dPG-(N_3_)_10%_–(COOH)_40%_6, with 40% of the hydroxyl groups functionalized as carboxylic acids (Fig. S7†).

### Design and characterization of 3D-NGs

Once the macromolecular precursors dPG-(BCN)_10%_4 and dPG-(N_3_)_10%_–(COOH)_80%_5 were synthesized, 3D-NGs displaying carboxylic acids could be prepared *via* inverse nanoprecipitation, which is characterized by the rapid formation of nanoparticles due to the fast mixing of a polymer solution in an organic solvent with a non-solvent. This method allows for precise control over particle size and distribution.^11,39^ To establish a method for the reproducible preparation of 3D-NGs 7, various parameters were evaluated, including the amount of solvent and non-solvent, reaction and quenching times, and stirring speeds. These conditions promote localized crosslinking within discrete nuclei and induce controlled phase separation, preventing growth into bulk gels and stabilizing nanogels as discrete particles. The quenching step effectively terminates further crosslinking, fixing the particle size, while stirring ensures homogeneous dispersion and prevents aggregation.^40^ 3D-NGs in a size range between 200–300 nm were targeted to meet the hypothesis of achieving multivalent binding interactions with *E. coli*. Both macromolecular precursors 4 and 5 were separately dissolved in MeOH, mixed in a syringe, injected into acetone under vigorous stirring, and quenched after 25 min with 3-azidopropanol. The resulting 3D-NG sizes, PDIs and ZPs are summarized in Table 1. The nanoprecipitation process was successfully scaled up to a batch size of 50 mg, producing 3D-NGs with a moderately broad size distribution of <0.25. Moreover, all 3D-NGs exhibited a strongly negative ZP, indicating the presence of carboxylic acids on the surface rather than being entangled within the polymer network. 3D-NG 7 of the 25 mg batch was further characterized using DLS, cryo-TEM, and ^1^H NMR (Fig. S8†) to determine particle size and morphology. DLS measurements showed a size of approximately 242 ± 9 nm and a ZP of −42 mV (Fig. 1B). Furthermore, 3D-NG 7 showed no change in size or ZP after being stored for 2 years at 4 °C in water, demonstrating stability under these conditions (Fig. 1C). Furthermore, cryo-TEM images confirmed the spherical shape of 3D-NG 7, composed of crosslinked dPG macromolecules (Fig. 1D).

**Table 1:** Size, PDI and ZP of 3D-NG 7 synthesis *via* inverse nanoprecipitation for different batch sizes

Batch size (mg)	MeOH (mL)	Acetone (mL)	Size^a^ (d.nm)	PDI	ZP (mV)
5	0.5	150	243 ± 3	0.05	−30 ± 2
25	1.0	750	242 ± 9	0.08	−42 ± 1
50	2.0	1500	245 ± 13	0.22	−29 ± 1

a In phosphate buffer (PB) (pH 7.4).

### Design of a 2D-graphene template

To create a comparative structure with the same number of carboxylic acids on the surface but a varying shape in spatial dimension, 2D-NGs were synthesized (Scheme S1†). Graphene was used as a platform for the synthesis as graphene surfaces are inert against usual reactions but can be functionalized in straightforward organic reactions.^33^ The process of cross-linker incorporation and activation is facilitated by graphene's π-conjugated system, while the colloidal graphene sheets maintain dispersibility in organic solvents.^33^ To avoid potential toxicity concerns of the graphene platform, the graphene template was subsequently removed after NG synthesis by cleavage of a pH-sensitive linker. Following template removal, the resulting 2D-NGs exhibit enhanced hydrophilicity and steric stabilization due to their crosslinked polymeric network, which leads to superior dispersibility and morphological control compared to the original 2D graphene template. For the comprehensive characterization of all graphene intermediates, a synergistic combination of analytical methods such as TGA, XPS, FTIR, and elemental analysis was used, each providing different and complementary information regarding size, morphology and composition.

**Fig. 2:**
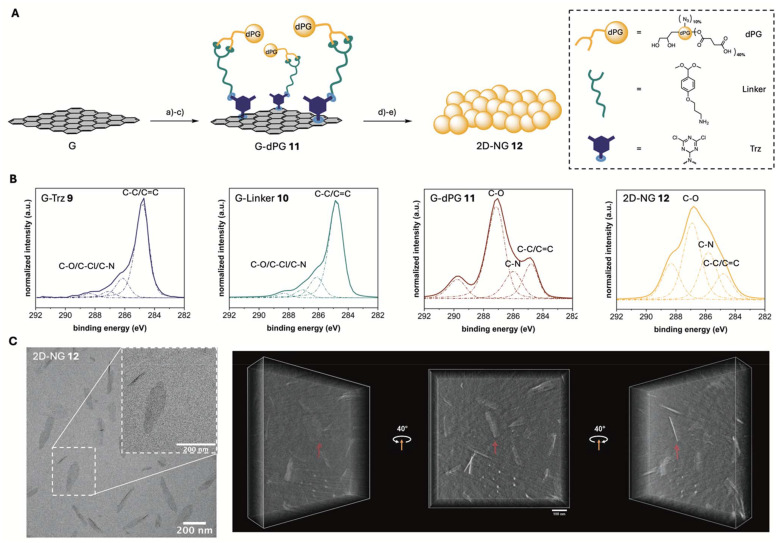
(A) Schematic illustration and synthetic route for 2D-NG 12. (a) cyanuric chloride, NaN_3_, NMP, 0 °C to 80 °C, 48 h; (b) DMPA, TEA, DMF, r.t., 48 h; (c) dPG-(N_3_)_10%_–(COOH)_40%_, PTSA, DMF, r.t., 24 h; (d) tripropargylamine, NaAsc, CuSO_4_·5H_2_O, DMF/H_2_O, 50 °C, 24 h; (e) 1 M HCl, r.t., 24 h. (B) Highly resolved C1s XP spectra of G-Trz 9, G-Linker 10, G-dPG 11, 2D-NG 12. The main components are denoted on each spectrum. For further details of the assigned components see Table S3.† (C) TEM image and TOMO of 2D-NG 12 in Milli-Q water at a concentration of 4 mg mL^−1^.

Thermally reduced graphene oxide (G) with a lateral size in the range of 200 nm to 1 μm was functionalized with dichlorotriazine groups *via* a nitrene [2 + 1] cycloaddition reaction to G-Trz 9 according to a published procedure (Scheme S1†).^33^ The density of functional groups on G-Trz 9 was 2.7% and 2.8%, determined by elemental analysis (Table S1†) and TGA (Fig. S9†) and calculated following a previously published procedure.^41^ In addition, an increased N1s content in XP survey spectrum (Fig. S10 and Table S2†) and appearance of new components ranging from 285.0 to 289.0 eV in the highly resolved C1s XP spectrum (Fig. 2B and Table S3†), attributed to the contribution of C–N and C–Cl bonds, proved covalent attachment of dichlorotriazine functional groups to the surface of G. The platform G-Linker 10 was synthesized through post-functionalization with DMPA, establishing pH-cleavable acetal bonds for covalent attachment to the diol groups of dPG (Scheme S1†).^42^ XP survey spectra revealed that G-Linker 10 was made up of carbon, nitrogen, and oxygen (Fig. S10†). The higher oxygen-to-carbon ratio in the XP survey spectrum, along with a reduced C–C/C=C component in the C1s XP spectrum and the decrease in nitrogen content in elemental analysis (Fig. 2B and Table S1†), indicated the effective attachment of DMPA to the surface of G-Trz 9. Afterwards, dPG-(N_3_)_10%_–(COOH)_40%_6 was conjugated to the linker by benzacetal moieties in the presence of catalytic amounts of PTSA to synthesize G-dPG 11 (Fig. 2A and Scheme S1†). Due to the dPG branches attached to the surface of G an oxygen peak at 530.0 eV in the XP survey spectrum of G-dPG 11 appeared (Fig. S10†). Additionally, high-resolution C1s XPS analysis revealed a significant intensity increase at 286.3 eV, characteristic of C–O bonds in dPG (Fig. 2B). The successful functionalization was further confirmed by TGA (Fig. S9†), showing 37% dPG content in G-dPG 11, and FTIR spectroscopy (Fig. S11†), exhibiting characteristic absorption bands at 1100, 2100, 2900, and 3400 cm^−1^, which are assigned to C–O, N_3_, aliphatic C–H, and OH bonds, respectively.

### 2D-NG synthesis and characterization

The crosslinking between the azide groups of dPG-(N_3_)_10%_–(COOH)_40%_6 and alkyne groups of tripropargylamine was performed by a CuAAC reaction (Scheme S1†). To avoid inter-template crosslinking, the crosslinker was adsorbed on the surface of G-dPG 11 and then released at 50 °C in a controlled manner to cause lateral crosslinking. This approach localizes the crosslinker near the polymer chains on the template surface, minimizing undesired network formation between templates and enabling selective crosslinking within individual 2D polymer sheets.^26,33^ Afterwards, the click-chemistry-derived 2D-dPG sheets were successfully released from the graphene template through selective cleavage of the acetal bonds under acidic conditions (Fig. 2A and Scheme S1†), with the complete template removal confirmed by both FTIR spectroscopy showing no characteristic graphene absorbance bands (Fig. S11†) and high-resolution C1s XPS analysis (Fig. 2B and Table S3†) revealing the disappearance of the C–C/C=C bond signal in the resulting 2D-NG 12. Furthermore, the strongly negative ZP of −32 mV observed for 2D-NG 12 indicated the presence of carboxyl groups on the NG surface.

Transmission electron microscopy (TEM) analysis of 2D-NGs 12 revealed a distinctive sheet-like structure (Fig. 2C). This morphological characteristic was further validated through tomographic investigations (Fig. 2C and Video S1†). Detailed dimensional analysis, conducted on a statistically sample size (*n* = 100), established a lateral size distribution of 213 ± 53 nm (Fig. S12†). The thickness of the material was precisely determined through grey value analysis (*n* = 10) and the tomographic investigation, yielding an average thickness of 5 ± 0.5 nm (Fig. 2C and Fig. S13†).

### Mannose functionalization of 2D- and 3D-NGs

The α-d-mannose (Man) functionalization of both 2D- and 3D-NGs was achieved through a controlled surface modification approach. While both systems are exclusively modifiable at their surfaces, they exhibit distinct functionalization patterns. For the 2D-NGs, the sheet-like structure provided an extended surface area for functionalization, allowing efficient attachment of mannose moieties to the accessible surface groups, resulting in a continuous distribution of functional groups across the surface. In contrast, the 3D-NGs offered a 3D network structure with surface modification sites, leading to a more patch-like distribution pattern of mannose groups, as only one of the two macromolecules provide groups for functionalization. This fundamental difference in the spatial arrangement of functional groups arises from the inherent structural features of both systems, which has a direct impact on their potential applications. The degree of functionalization (DF) with α-d-mannose was established at 40%, as this level demonstrated significantly stronger binding to various lectins compared to lower functionalization levels. Moreover, no substantial increase in binding was noted with higher degrees of functionalization, indicating that 40% is optimal for enhancing interaction with lectins.^43^

**Fig. 3:**
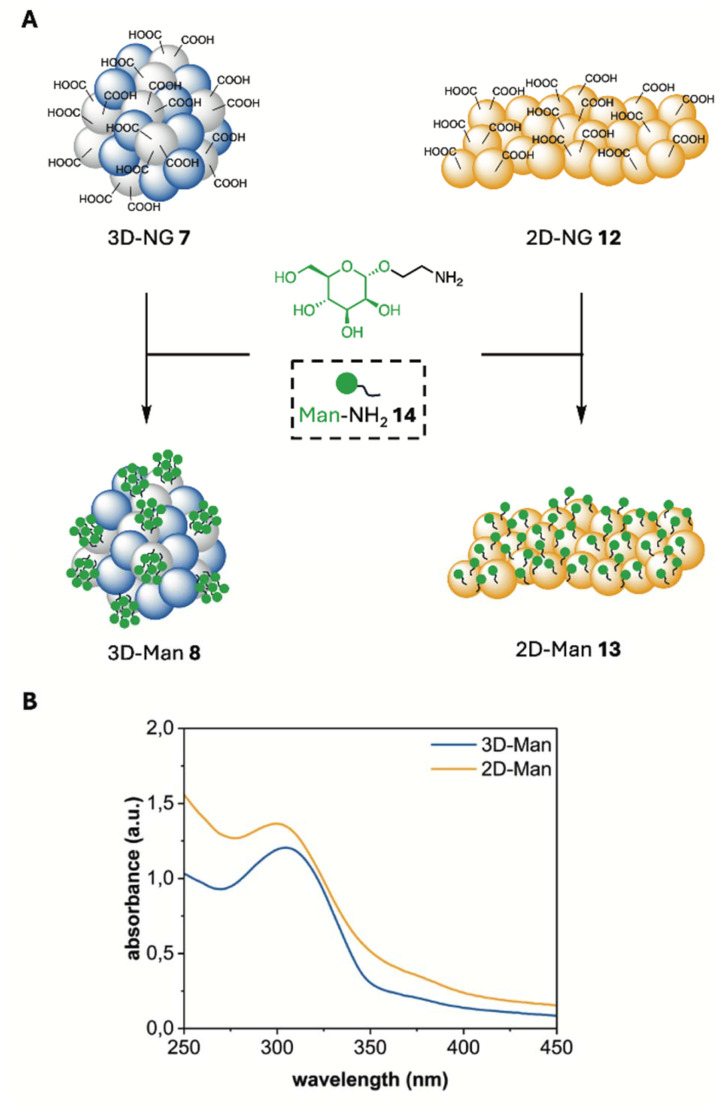
(A) Synthesis of mannose functionalized NGs. Reagent and conditions: DIPEA, HSTU, DMF, r.t., 36 h. (B) UV-Vis spectrum of 3D-Man 8 and 2D-Man 13 15 min after treatment with conc. H_2_SO_4_.

The surface functionalization of both 2D- and 3D-NGs was achieved through a controlled amide coupling strategy. The key mannose derivative, Man-NH_2_14, was synthesized following an established procedure.^35^ Prior to the coupling reaction, NGs 7 and 12 were transferred into DMF to ensure optimal reaction conditions and prevent potential hydrolysis of the activated species. Carboxylic acid groups were activated using DIPEA and HSTU, forming active NHS-esters *in situ* under argon atmosphere. The addition of Man-NH_2_14 and the subsequent purification by dialysis yielded 3D-Man 8 and 2D-Man 13 (Fig. 3A). The successful surface modification was confirmed by DLS measurements and ZP analysis (Table 2). For 3D-Man 8, an increase in hydrodynamic diameter to 265 ± 14 nm was observed, accompanied by a slight increase in PDI to 0.25. Similarly, 2D-Man 13 exhibited a particle size of 194 ± 48 nm as determined by TEM. The ZP of 3D-Man 8 significantly changed from −42 mV to −11 mV. Similarly, 2D-Man 13 showed a ZP change from −32 mV to −13 mV. These changes strongly support successful functionalization as the reduction in negative ZP values indicates the conversion of carboxylic acid groups to neutral amide bonds, while the increase in particle size for 3D-Man 8 can be attributed to the addition of the mannose layer. The moderate increase in PDI is consistent with surface modification, as functionalization typically introduces some heterogeneity to the particle population. Furthermore, electron microscopy of 3D-Man 8 and 2D-Man 13 revealed no structural differences between the systems, confirming that both NG architectures maintain their integrity after mannose modification (Fig. S18 and S19†).

**Table 2:** DLS results of 3D-Man 8 and 2D-Man 13 synthesis and the theoretical (theor.) and experimental (exp.) amount of mannose (wt.%) in Man-NGs calculated with the H_2_SO_4_/UV-Vis assay

	Size^a^ (d.nm)	ZP (mV)	% Man (theor.)	% Man (exp.)
3D-Man 8	265 ± 14	−11	40.0	31.7
2D-Man 13	194 ± 48	−13	40.0	34.0

a In PB, pH 7.4.

The α-d-mannose surface density was determined using the H_2_SO_4_/UV-Vis assay described by Albalasmeh *et al.* (Fig. 3B, Table 2 and Fig. S17†).^36^ 3D-Man 8 showed an experimental mannose content of 31.7 wt%, while the theoretical calculation suggested a value of 40 wt%. Similarly, 2D-Man 13 achieved an experimental mannose content of 34.0 wt% with the same theoretical calculation of 40 wt%. It is important to emphasize that mannose functionalization was partial in both NG systems, as the experimental values fall short of the theoretical maximum. This incomplete modification likely arises from steric hindrance and limited accessibility of reactive sites, which differ between the 3D crosslinked network and the 2D monolayer architecture, reflecting their structural characteristics and spatial arrangements of functional groups.

3D-NG 7 was synthesized through three-dimensional crosslinking of macromolecules in solution, creating a spatially distributed network structure. This 3D assembly process may affect the distribution of carboxyl groups within the NG matrix, influencing their availability for mannose coupling reactions. In contrast, 2D-NG 12 was synthesized using a template-directed approach, resulting in a monolayer architecture. This controlled 2D assembly method promotes the spatial arrangement of carboxyl groups predominantly on the surface, rather than within the NG structure.

Following the mannose functionalization of both 2D- and 3D-NGs, it is important to consider how their distinct morphologies influence binding behavior. Although a precise quantitative comparison of the specific surface areas within the same volume was not feasible in this study, comparable sample volumes and concentrations were employed to enable a relative assessment primarily driven by morphological differences. The sheet-like 2D-NGs 12 offer an extended planar surface that facilitates ligand presentation, while the more compact 3D-NGs 7 form volumetric networks which may limit accessibility but enhance multivalent interactions.

### Evaluating the performance of Man-NGs as lectin binders

To evaluate the biological activity and accessibility of mannose ligands on the NGs, their interaction with the model lectin Concanavalin A (ConA) was studied. ConA specifically recognizes terminal α-d-mannose residues and, upon binding to multivalent glycans, mediates the formation of extensive cross-linked aggregates.^44^ This aggregation process leads to increased sample turbidity due to light scattering by the growing complexes. We monitored this turbidity increase (absorbance at 340 nm) as a measure of the ConA interaction kinetics and extent, following established methodologies for studying lectin-glycan interactions *via* turbidimetry.^45^ The aim was to investigate the influence of spatial ligand presentation by comparing the ConA binding behavior of systems with distinct architectures: spherical 3D-Man-NGs, sheet-like 2D-Man-NGs, and yeast mannan (YMan) as a dissolved polymer reference.

**Fig. 4:**
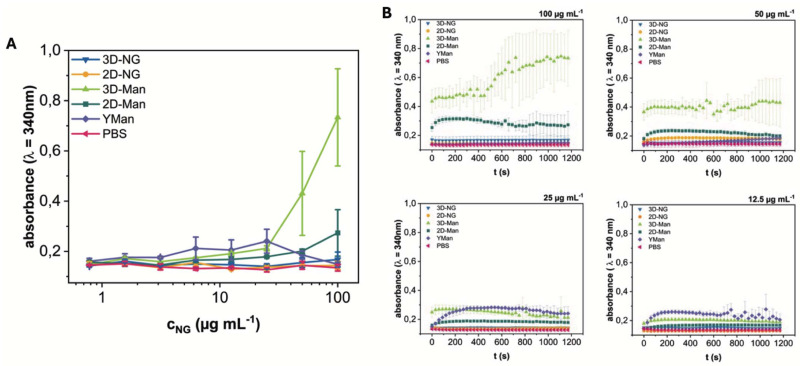
(A) Aggregate formation by ConA (*c* = 150 μg mL^−1^) and varying concentrations of NGs (3D-NG, 2D-NG, 3D-Man, and 2D-Man) and yeast mannan (YMan), as determined by turbidimetry. End point values after 20 min. Further informations are assigned in Fig. S18.† (B) Kinetics of the binding and aggregate formation of ConA with NG samples and controls. Plotted are the mean absorbance values at 340 nm ± SD over time from 3 independent measurements.

Turbidity measurements revealed concentration-dependent aggregation profiles for the different systems (Fig. 4A and Fig. S20†). YMan exhibited a turbidity profile resembling the classical precipitin reaction with maximal turbidity observed between 12.5–25 μg mL^−1^ and a corresponding decrease in ConA concentration in the supernatant (Fig. S22†).^46^ At higher and lower concentrations, turbidity was decreased. This bell-shaped curve is characteristic of multivalent interactions where optimal lattice formation occurs near equivalence; the subsequent decrease in turbidity at higher glycan concentrations is primarily attributed to the prozone effect, where excess glycan inhibits extensive cross-linking, leading to smaller, less light-scattering complexes.

In contrast, both 3D-Man 8 and 2D-Man 13 showed continuously increasing turbidity with increasing concentration, eventually reaching a plateau at higher concentrations, without the optimum and subsequent decline observed for YMan. Notably, 3D-Man 8 consistently yielded higher turbidity levels than 2D-Man 13 across the tested range. Control experiments with non-mannosylated 3D-NG 7 and 2D-NG 12 showed no significant turbidity increase, confirming the specificity of the mannose-ConA interaction (Fig. S21†). Additional studies using varying ConA concentrations at fixed NG concentrations (*c* = 80 μg mL^−1^) revealed saturation behavior at a ConA concentration of about 3 mg mL^−1^ (Fig. S21†).

The plateauing behavior observed for the NGs indicates that a prozone effect causing turbidity decrease was not operative within the tested concentration range. This difference compared to YMan likely stems from the distinct nature of the NGs as pre-formed colloidal particles rather than dissolved polymers. Plausible reasons include high-avidity interactions driven by the dense surface presentation of mannose on the NGs being less susceptible to inhibition by excess particles, or steric constraints imposed by the NG structure hindering the formation of the smaller, soluble complexes characteristic of the prozone effect. Within this context of particle aggregation, the higher turbidity plateau reached by 3D-Man 8 compared to 2D-Man 13, despite similar mannose content, suggests that the spherical 3D architecture facilitates more extensive ConA-mediated aggregation, possibly due to enhanced ligand accessibility or optimal spatial arrangement for inter-particle bridging.

Analysis of the aggregation kinetics revealed concentration-dependent rates of turbidity increase (Fig. 4B). For the highest concentrations tested, turbidity generally saturated after approximately 200 s. At later time points (>500 s), signal noise increased and some complex fluctuations were observed. Consistent with the endpoint turbidity levels, 3D-Man 8 induced the fastest initial rate of turbidity increase, followed by 2D-Man 13. Both NG systems exhibited faster initial aggregation kinetics compared to the dissolved YMan polymer. These kinetic differences further support the notion that the spatial presentation of mannose on the pre-formed NG structures, particularly the 3D architecture, allows for more efficient initial cross-linking by ConA.

In order to investigate binding affinities to intact *E. coli* ORN 178 (FimH^+^), the dissociation constant (*K*_D_) was determined by microscale thermophoresis (MST).^47^ The technique makes use of molecular migration due to an applied temperature gradient and is sensitive to changes in molecular size, hydration shell, and charge, and can thus detect binding interactions in complex biological systems, including multivalent interactions.^48^ Binding affinities are evaluated from the changes in the thermophoretic behaviour resulting from binding of the ligand to the protein.^49^ Our system represents multivalent binding (overall avidity), as both the bacterial surface and the NGs present multiple binding sites – mannose residues on the NGs and lectin-like receptors (FimH) on the bacterial surface.

**Fig. 5:**
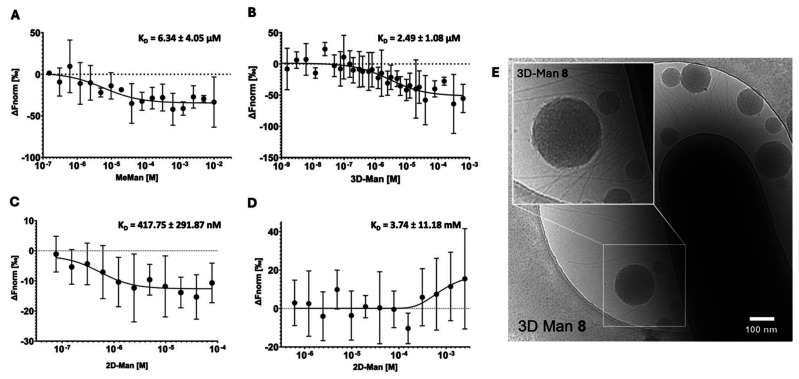
MST of (A) MeMan, (B) 3D-Man, (C) 2D-Man (first binding site), and (D) 2D-Man (second binding site) titrated against *E. coli* ORN178 (FimH^+^). Each data point represents biological repeats of *n* = 6. (E) Cryo-TEM image shows mannose-dependent 3D-Man binding to *E.coli* ORN178 (FimH^+^).

The non-mannosylated 3D-NG 7 and 2D-NG 12 (negative controls) showed randomly fluctuating values for the MST response throughout the ligand titration range (Fig. S23†). However, similar to α-d-mannopyranoside (MeMan, positive control), optimised binding behaviour due to multivalent display of the mannose ligands on 3D-Man 8 and 2D-Man 13 was observed. In the presence of 3D-Man 8 and 2D-Man 13, characteristic s-shaped and mirrored-s-shaped sigmoidal binding curves could be obtained. The different appearances of the binding curves can be attributed to different thermophoretic movements of the respective compounds.^49^ Quantitative comparison of the binding data revealed distinct patterns in the efficiency of multivalent interactions. The high-affinity binding of 2D-Man 13 (*K*_D_ = 0.42 μM) (Fig. 5C) showed a 6-fold stronger interaction compared to 3D-Man 8 (*K*_D_ = 2.49 μM) (Fig. 5B) in absolute terms. This difference was maintained after mannose normalization, with the high-affinity site of 2D-Man 13 (*K*_D_ = 1.23 μM) exhibiting approximately 6.4-fold stronger binding than 3D-Man 8 (*K*_D_ = 7.85 μM). The normalized binding affinity of 3D-Man 8 closely matched that of the monovalent MeMan reference (*K*_D_ = 6.34 μM) (Fig. 5A). The low-affinity site of 2D-Man 13 (Fig. 5D) showed substantially weaker binding both before (*K*_D_ = 3740 μM) and after normalization (*K*_D_ = 11 000 μM), with binding constants three to four orders of magnitude higher than those of the other interactions measured. These quantitative differences in binding affinities likely result from the differences in ligand presentation: the planar distribution of mannose residues on 2D-Man 13 may enable more simultaneous interactions with the FimH receptors on the bacterial surface, enhancing multivalent binding, whereas the more globular arrangement in 3D-Man 8 may limit such cooperative interactions due to steric hindrance or suboptimal spatial alignment. All in all, the measurements demonstrate that the spatial presentation of binding sites significantly influences the measured binding affinities to FimH on intact *E. coli* ORN 178.

In addition, cryo-TEM images of 3D-Man 8 were taken (Fig. 5E). The mannosylated NGs showed clear co-localization to the bacteria and their pili, supporting the results of the binding assays. Due to low contrast, 2D-Man 13 could not be reliably visualized by cryo-TEM despite attempts. Thus, cryo-TEM data serve as complementary evidence alongside the biological assays.

## Conclusions

In conclusion, the successful design and synthesis of two distinct architectures of mannosylated nanogels with well-defined structural characteristics and biological functionality has been demonstrated. The 3D-NG system was prepared *via* nanoprecipitation, while the 2D-NG system was synthesized using a graphene-template strategy with subsequent template removal. Both NGs were comprehensively characterized using multiple analytical techniques, confirming their intended architectures, sizes, and surface modifications. The 3D-NGs exhibited remarkable stability over two years, while both systems maintained their structural integrity after mannose functionalization. Turbidity measurements with ConA revealed distinct aggregation behavior for both architectures, with 3D-Man showing enhanced aggregation kinetics compared to 2D-Man. Binding studies with FimH on intact *E. coli* strain ORN 178 revealed interaction profiles for the different architectures. 3D-Man exhibited single-site binding behavior with affinity comparable to the monovalent reference compound. In contrast, 2D-Man demonstrated dual binding behavior with a high-affinity and a low-affinity binding site, separated by several orders of magnitude in binding strength. The high-affinity site of 2D-Man maintained superior binding compared to 3D-Man. Cryo-TEM imaging provided evidence for specific binding interactions, with the 3D-Man system showing clear co-localization with bacterial pili. These results show that the spatial arrangement of the mannose units significantly influences multivalent binding characteristics, even if a similar mannose content is present in the systems. This work provides valuable insights into the design principles for multivalent glyco-architectures and their impact on bacterial adhesion, offering promising strategies for developing novel anti-adhesive therapeutics and surface modifications.

## Author contributions

Coceptualization, investigation, validation, visualization, formal analysis, data interpretation, methodology, writing – original draft: A.-C. S. Conceptualization, investigation, data interpretation, validation: M. B. Conceptualization, methodology, investigation, formal analysis, data interpretation, validation, *in vitro* studies, writing – review and editing: S. W. Investigation, conceptualization, writing – review and editing: M. D. Investigation, formal analysis: P. N. Investigation, formal analysis, writing – review and editing: K. L. Conceptualization, methodology, investigation, formal analysis, data interpretation, validation, *in vitro* studies, writing – review and editing: T. L. P. Conceptualization, methodology, supervision, project administration, resources, funding acquisition, writing – review and editing: R. H.

## Conflicts of interest

There are no conflicts to declare.

## Supplementary Material

BM-013-D5BM00286A-s001

## Data Availability

The data that support the findings of this study are available in the ESI.† Additional data can be accessed from the corresponding authors upon reasonable request.
