# Supplementary material for: Multivalent 2D- and 3D-nanogels as carbohydrate-lectin binders
Source: Biomater Sci. 2025 Jul 7;13(16):4400–11. doi: 10.1039/d5bm00286a (PMC12230938; doi:10.1039/d5bm00286a)
Supplement: BM-013-D5BM00286A-s001 [file BM-013-D5BM00286A-s001.pdf]

Supplementary Information (SI) for Biomaterials Science.
This journal is © The Royal Society of Chemistry 2025

Supplementary Information

Multivalent 2D- and 3D-nanogels as carbohydrate-lectin binders†

Ann-Cathrin Schmitt,[a] *Maximilian Braun,*[a] *Stefanie Wedepohl,*[b] *Mathias Dimde,*[c] *Philip Nickl,*[b] *Kai Ludwig,*[c] *Tatyana L. Povolotsky,**[b] *Rainer Haag**[a,b]

[a] Institute of Chemistry and Biochemistry, Freie Universität Berlin, Takustr. 3, 14195 Berlin, Germany.

[b] SupraFAB, Freie Universität Berlin, Altensteinstr. 23a, 14195 Berlin, Germany.

[c] Forschungszentrum für Elektronenmikroskopie (FZEM), Freie Universität Berlin, Fabeckstr. 36A, 14195 Berlin, Germany.

Methods:

Fourier-transform infrared spectroscopy (FTIR). FTIR measurements were performed on a JASCO FT/IR spectrometer (JASCO Deutschland GmbH, Pfungstadt, Germany) via attenuated total reflectance (ATR). Wave numbers $\tilde{\upsilon}$ are given in cm-1.

Thermal gravimetric analysis (TGA). TGA measurements were recorded on Linseis STA PT1600 and evaluated with Linseis Data Acquisition software. The measurements conducted at temperatures ranging from 25 °C to 800 °C with 10 °C/min heating rate under air atmosphere. Measurements were performed in Al2O3 crucibles. Sample masses varied from 7 to 15 mg.

Synthetic procedures:

dPG-(OMs)$_{10\%}$ 1a. This reaction was performed according to a modified procedure.[1] To a solution of dPG-OH (5.00 g, 0.50 mmol, 1.0 eq.) in anhydrous DMF (50 mL), triethylamine (TEA) (1.60 mL, 8.40 mmol, 1.2 eq. per OH group) was added. The solution was cooled down to 0 °C. After stirring at 0 °C, methane sulfonyl chloride (MsCl) (0.65 mL, 8.40 mmol, 1.2 eq per OH group) was added dropwise via a syringe and the reaction mixture was stirred at r.t. for 24 h. The mixture was filtered, the solvent was evaporated under reduced pressure and the crude product was dialyzed against DMF for 24 h (MWCO 2 kDa). The yellow viscous product dPG-(OMs)$_{10\%}$ **1** (4.77 g, 0.43 mmol, 10% conversion, 87%) was dried overnight.

^{1}H NMR (500 MHz, D_2O) *δ* (ppm): 4.20 – 3.20 (m, 5H, polyglycerol backbone), 3.26 (t, 3H, H4).

dPG-(N_3)$_{10\%}$ 2. This reaction was performed according to a modified procedure.[1] dPG-(OMs)$_{10\%}$ **1a** (4.00 g, 0.36 mmol, 1.0 eq.) was dissolved in anhydrous DMF (40 mL) under an inert gas atmosphere and exclusion of water. After adding NaN_3 (0.98 g, 15.12 mmol, 3.00 eq. per OMs group), the resulting suspension was heated to 60 °C and stirred for 72 h. After cooling, filtration delivered a yellowish filtrate and a white residue of excess NaN_3. The filtrate was concentrated under reduced pressure and the product was dialyzed (MWCO 2 kDa) against MeOH and H_2O for 24 h, respectively. The product dPG-(N_3)$_{10\%}$ **2** (2.38 g, 0.23 mmol, quant. conversion, 65%) was dried overnight.

^{1}H NMR (500 MHz, D_2O) *δ* (ppm)): 4.20 – 3.20 (m, 5H, polyglycerol backbone (H1-H3)).

FTIR (ATR, cm^{-1}): 3600 – 3200 (n(OH)), 3550 – 3350 (n(NH_2)), 3000 – 2850 (n(CH, CH_2, CH_3)), 1650 (d(NH_2)).

dPG-(NH$_2$)$_{10\%}$ 3. This reaction was performed according to a modified procedure.[2] dPG-(N$_3$)$_{10\%}$ **2** (0.50 g, 0.05 mmol, 1.0 eq.) was dissolved in H_2O and MeOH. Tris(2-chlorethyl) phosphate (TCEP) (0.28 g, 1.02 mmol, 1.50 eq. per N$_3$-group) was added and stirred for 24 h at r.t. The resulting solution was dialyzed against MeOH for 24 h (MWCO 2 kDa). The product dPG-(NH$_2$)$_{10\%}$ **3** (0.43 g, 0.04 mmol, 85% yield) was stored in MeOH.

FTIR (ATR, cm^{-1}): 3600 – 3200 (n(OH)), 3000 – 2850 (n(CH, CH$_2$, CH$_3$)), 2100 (n(N$_3$)).

dPG-(BCN)$_{10\%}$ 4. Bicyclo[6.1.0]non-4-yn-9-ylmethyl-(4-nitrophenyl) carbonate (BCN) was synthesized according to a published procedure and subsequently coupled to dPG (NH$_2$)$_{10\%}$ **3** according to a modified procedure.[3, 4] To a solution of dPG-(NH$_2$)$_{10\%}$ **3** (0.20 g, 0.25 mmol, 1.0 eq.) in anhydrous DMF (10 mL), triethylamine (TEA) (50 µL, 0.37 mmol, 1.5 eq.) and BCN (77.00 mg, 0.25 mmol, 1.0 eq.) were added. The reaction mixture was stirred for 24 h at r.t. and the solvent was removed under reduced pressure. The crude product was dialyzed against MeOH for 24 h (MWCO 2 kDa) and the dialysate was changed regularly. The product dPG-(BCN)10% 4 (0.17 g, 12.00 µmol, quant. conversion, 68%) was stored in MeOH.

^{1}H-NMR (500 MHz, D$_2$O, δ (ppm)): 4.50 – 3.30 (m, 5H, polyglycerol backbone (H1-H3)), 2.55 – 0.75 (m, BCN moieties (H4-H9)).

Synthetic route of 2D-NG **12**:

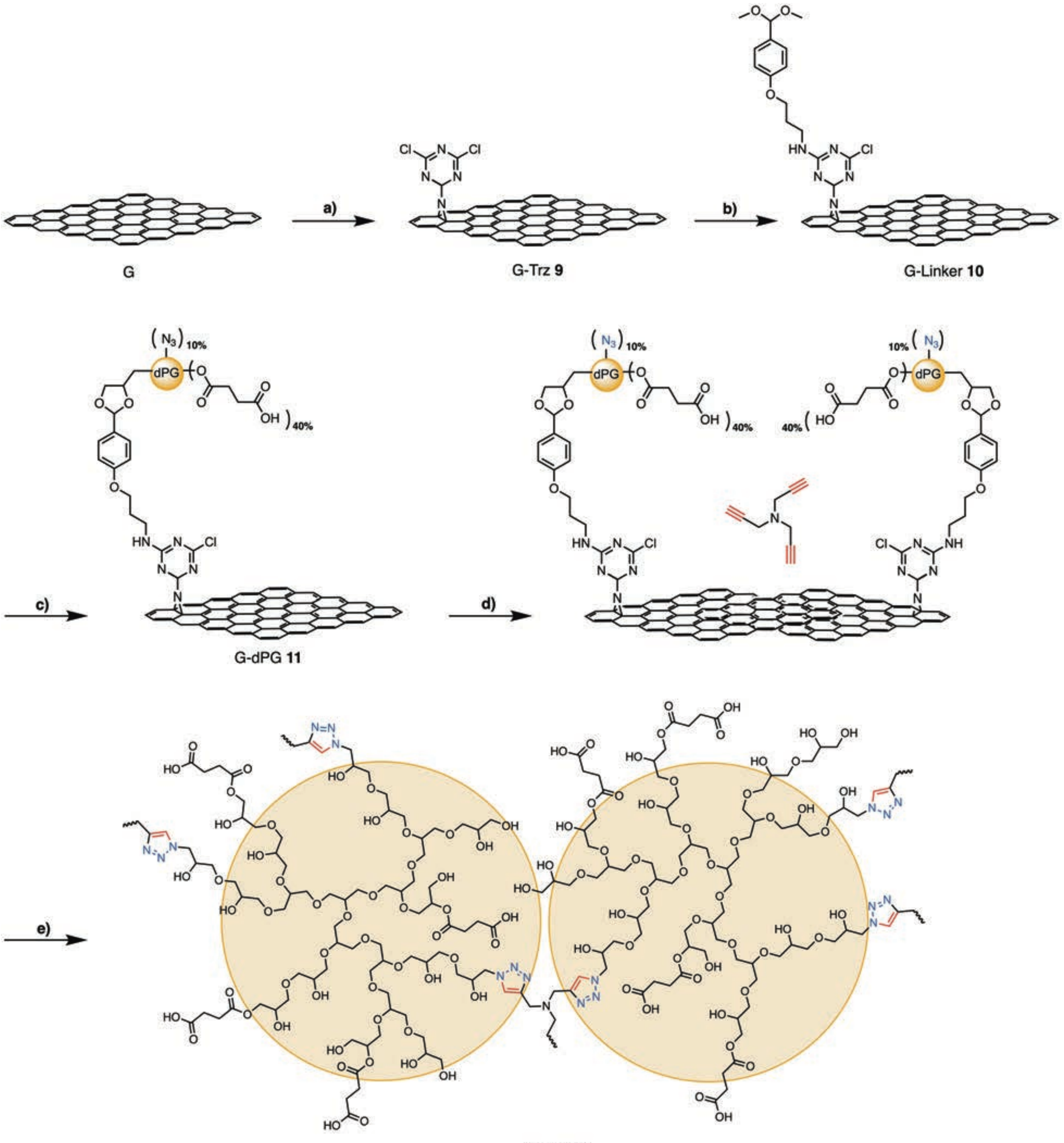

Scheme S1. Synthetic route of 2D-NG **12**. Reagents and conditions: a) Cyanuric chloride, NaN_3, NMP, 0 °C to 80 °C, 48 h. b) DMPA, TEA, DMF, r.t., 48 h. c) dPG-$(N_3)_{10\%}$-$(COOH)_{40\%}$, PTSA, DMF, r.t., 24 h. d) Tripropargylamine, NaAsc, $CuSO_4 \cdot 5H_2O$, DMF/H_2O, 50 °C, 24 h. e) 1 M HCl, r.t., 24 h.

G-Trz 9. 280 mg of thermally reduced graphene oxide (TRGO) were dispersed in 100 mL of dry N-methyl-2-pyrrolidone (NMP) and cooled to 0 °C. Cyanuric chloride (10.0 g, 54 mmol, 1.0 eq.) was dissolved in 25 mL NMP, cooled down to 0°C and subsequently sodium azide (3.5 g, 54 mmol, 1.0 eq.) was slowly added and stirred for 45 min at 0 °C. Afterwards the solution was added to the TRGO dispersion and the mixture was stirred for 2 h at 0 °C and for 24 h at 80 °C. Meantime, the solution was sonicated and argon was bubbled into the solution. Centrifugation was then carried out at 10,000 rpm for 45 min and the resulting precipitated product was washed four times each with THF, acetone and water. After each step, the solution was sonicated for 20 min and centrifugated (30 min, 10,000 rpm). Finally, the black product G-Trz **9** was lyophilized (478.3 mg).

G-Linker 10. G-Trz **9** (35 mg) was dispersed in dry DMF (35 mL) for 30 min in an ultrasonic bath. Afterwards, 3-(4-(dimethoxymethyl)phenoxy)propan-1-amine (DMPA) (270,0 mg, 1.20 mmol, 1.0 eq.) dissolved in dry DMF and 120 μL of TEA (0,84 mmol, 0.7 eq.) were added to the solution. The mixture was stirred for 48 h at room temperature. The black product G-Linker **10** (35.7 mg) was obtained after 2 days dialysis (MWCO 2 kDa) against MeOH.

(2-aminoethyl)-α-D-mannopyranoside (Man-NH_2) 14. Man-NH_2 **14** was synthesized according to a previously published procedure.[5] (2-azidoethyl)-α-D-mannopyranoside (100.0 mg, 0.40 mmol, 1 equiv.) was dissolved in 5 mL methanol. The solution was bubbled with argon for 2 min. Pd/C (12.0 mg, 10 wt.% loading) was then added and the mixture was stirred for 24 h at room temperature under a hydrogen atmosphere. The catalyst Pd/C was filtered over celite, and the resulting colorless solution was washed twice with methanol. The product Man-NH_2 **14** (86.1 mg, 0.38 mmol, 96%) was obtained by removing the solvent under vacuum.

^{1}H NMR (500 MHz, MeOH-d_4, δ (ppm)): 4.77 (d, 1H, H3), 3.86 – 3.76 (m, 3H, H5, H8), 3.72 – 3.44 (m, 5H, H2, H4, H6, H7), 2.82 (m, 2H, H1).

^{13}C NMR (500 MHz, MeOH-d_4, δ (ppm)): 101.76 (C3), 74.74 (C7), 72.59 (C4), 72.08 (C5), 69.97 (C6), 68.65 (C2), 62.95 (C8), 42.06 (C1).

FTIR (ATR, cm^{-1}): 3600 – 3200 (n(OH)), 3550 – 3350 (n(NH_2)), 1650 (d(NH_2)).

Supporting Figures:

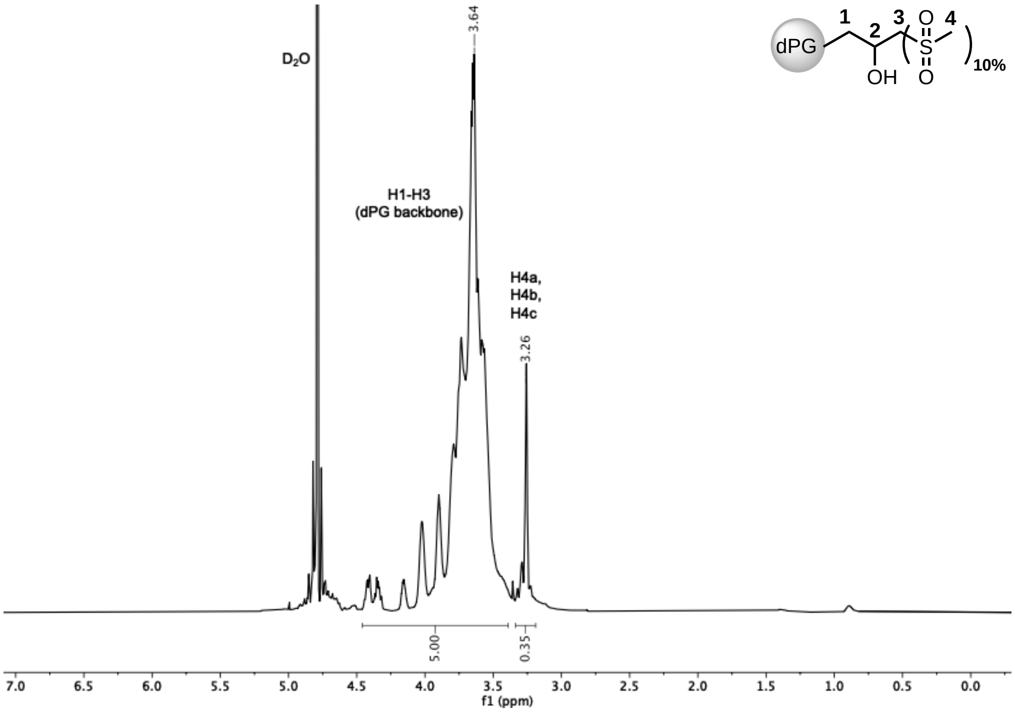

Figure S1. ^{1}H NMR spectrum of dPG-(OMs)$_{10\%}$ **1a** recorded in D_2O.

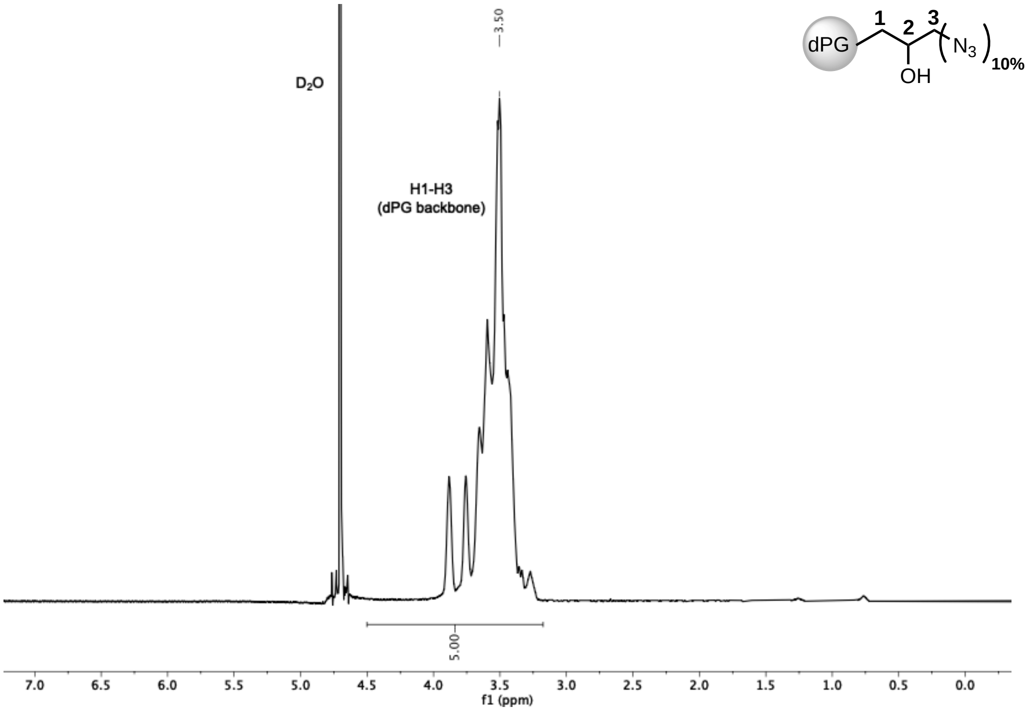

Figure S2. ^{1}H NMR spectrum of dPG-$(N_3)_{10\%}$ **2** recorded in D_2O.

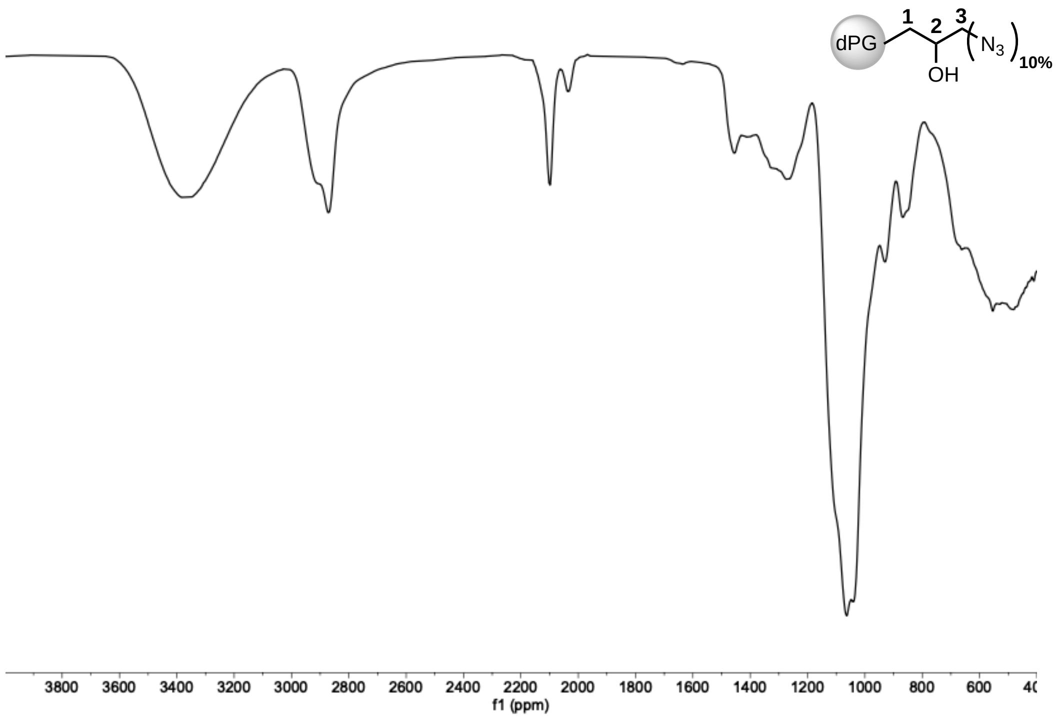

Figure S3. FTIR spectrum of dPG-$(N_3)_{10\%}$ **2**.

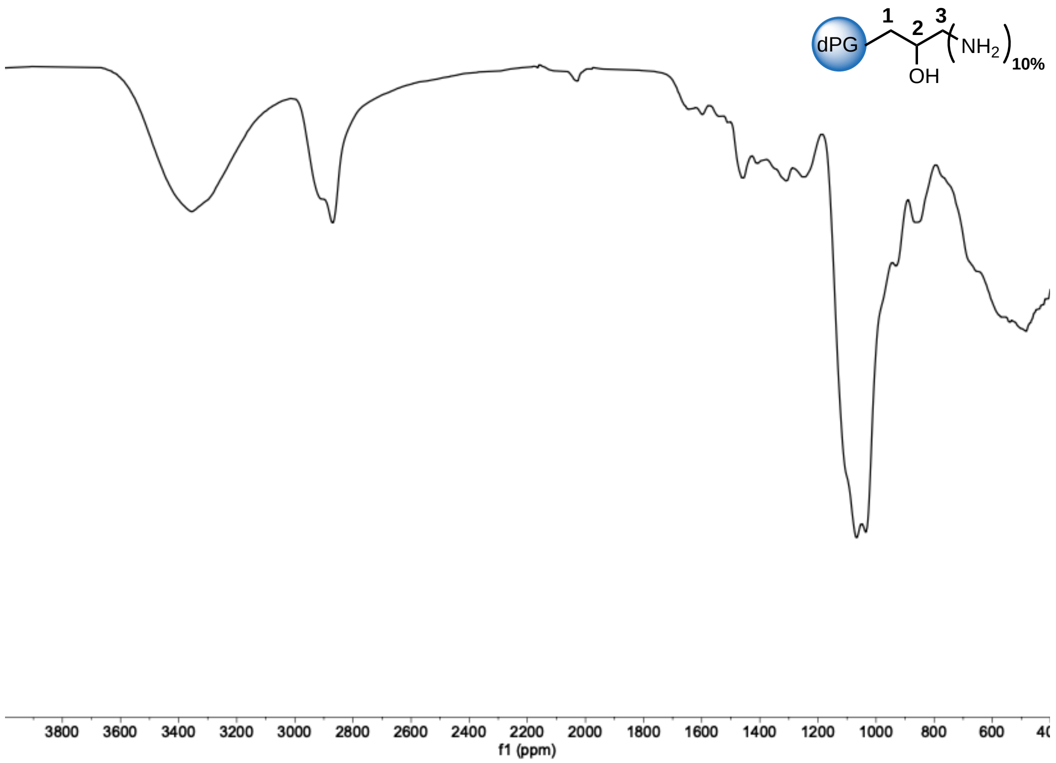

Figure S4. FTIR spectrum of dPG-$(NH_2)_{10\%}$ **3**.

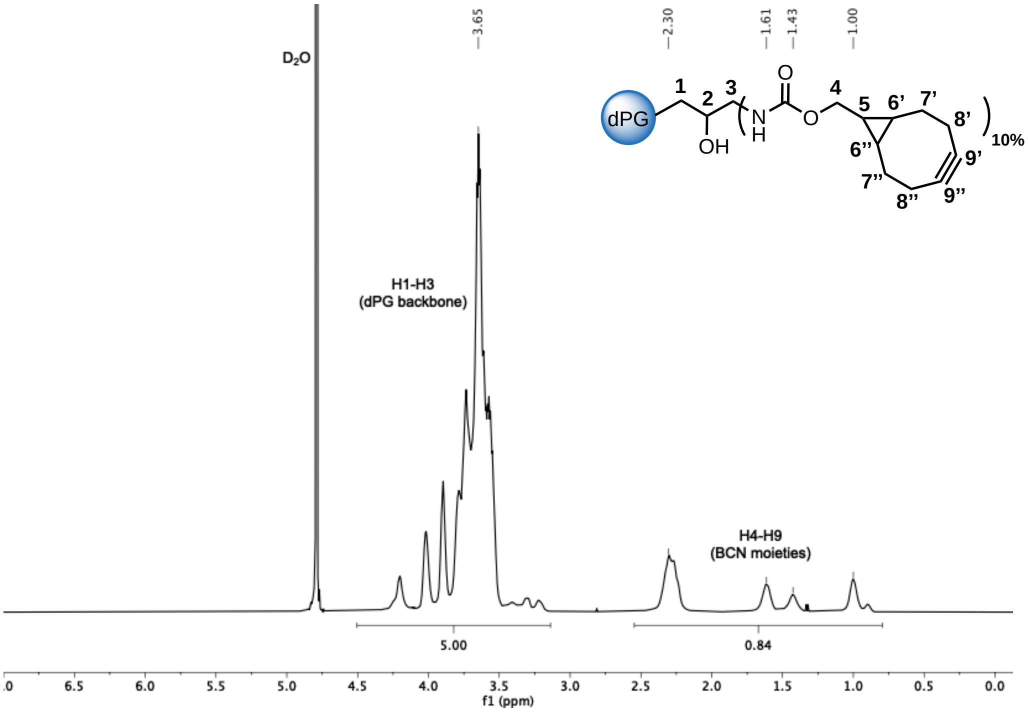

Figure S5. ^{1}H NMR spectrum of dPG-(BCN)$_{10\%}$ **4** recorded in D_2O.

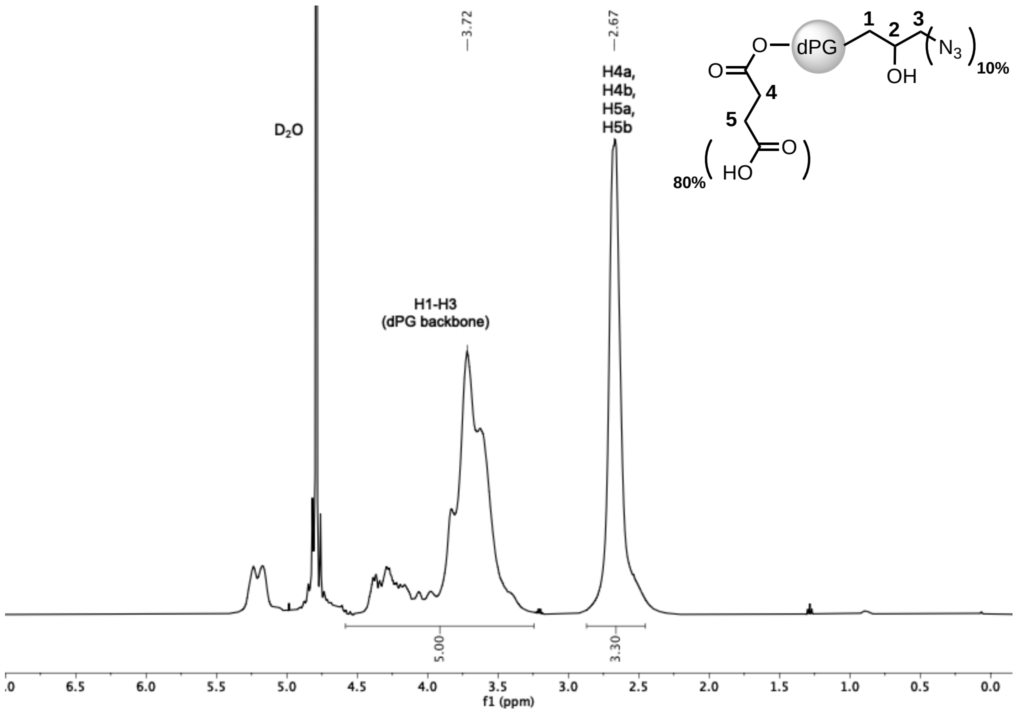

Figure S6. ^{1}H NMR spectrum of dPG-$(N_3)_{10\%}$-$(COOH)_{80\%}$ **5** recorded in D_2O.

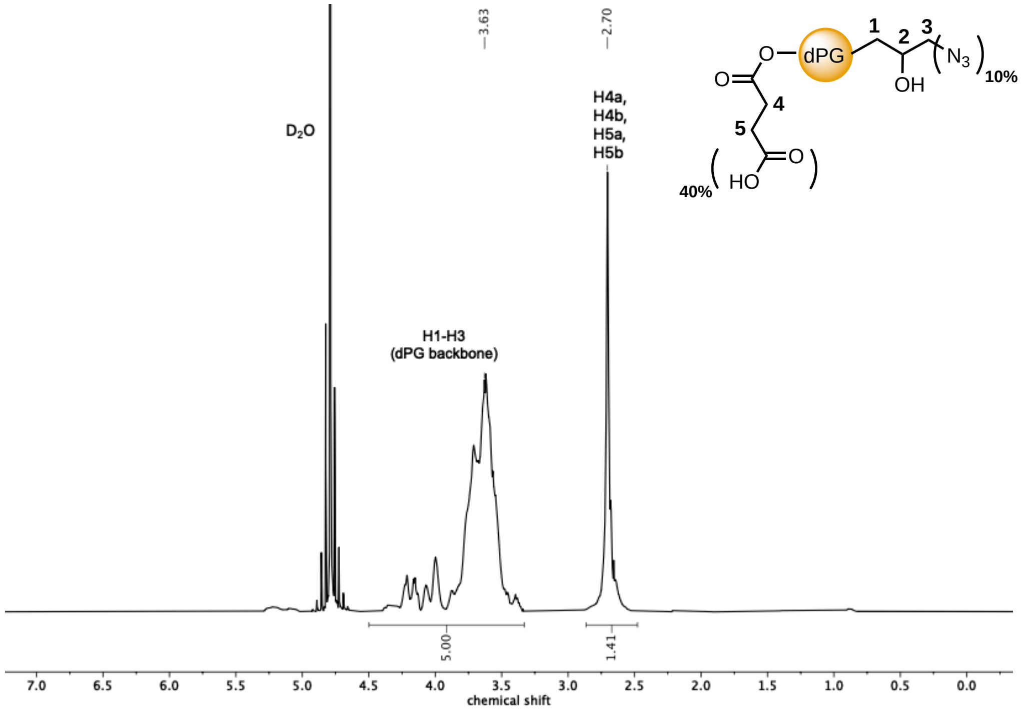

Figure S7. ^{1}H NMR spectrum of dPG-$(N_3)_{10\%}$-$(COOH)_{40\%}$ **6** recorded in D_2O.

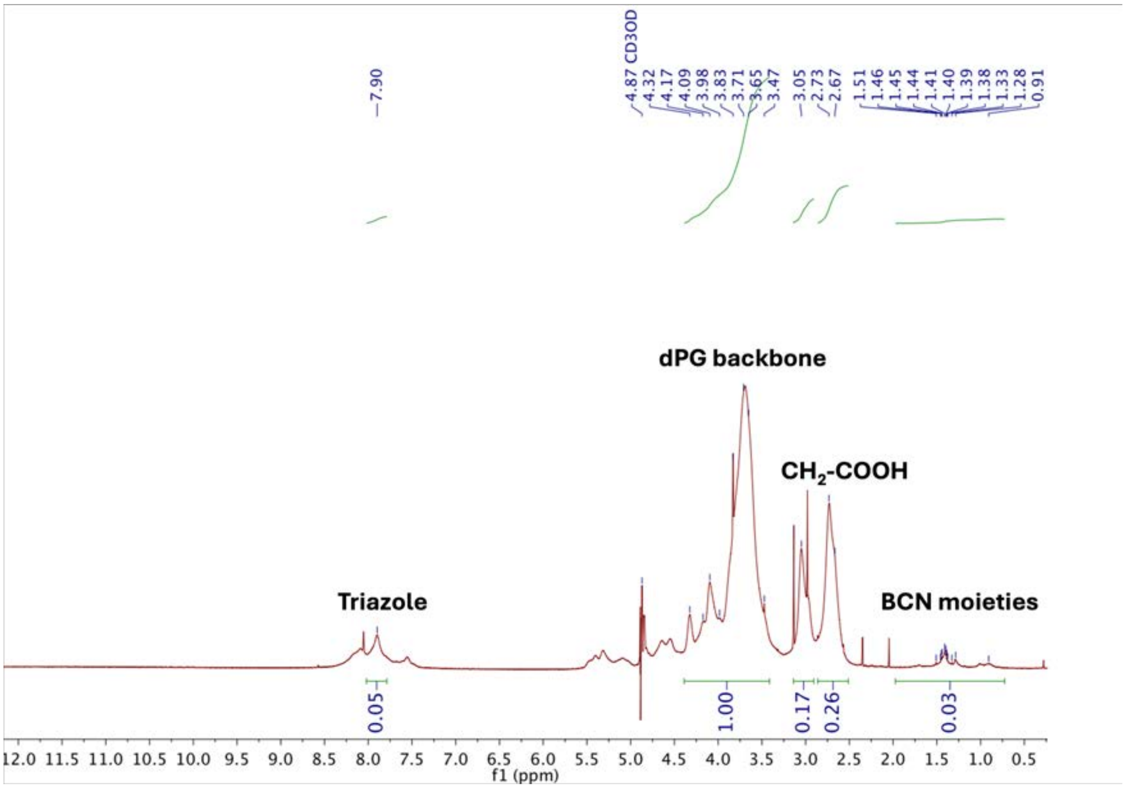

Figure S8. ^{1}H NMR spectrum of 3D-NG **7** recorded in D_2O.

Table S1. Elemental analysis of G-Trz, G-Linker, G-dPG and 2D-NG.

Sample	C in %	H in %	N in %	S in %
G	84.6	1.2	-	-
G-Trz	74.2	2.5	8.2	-
G-Linker	73.8	2.7	6.9	-
G-dPG	61.8	6.5	5.2	-
2D-NG	57.2	6.6	5.0	-

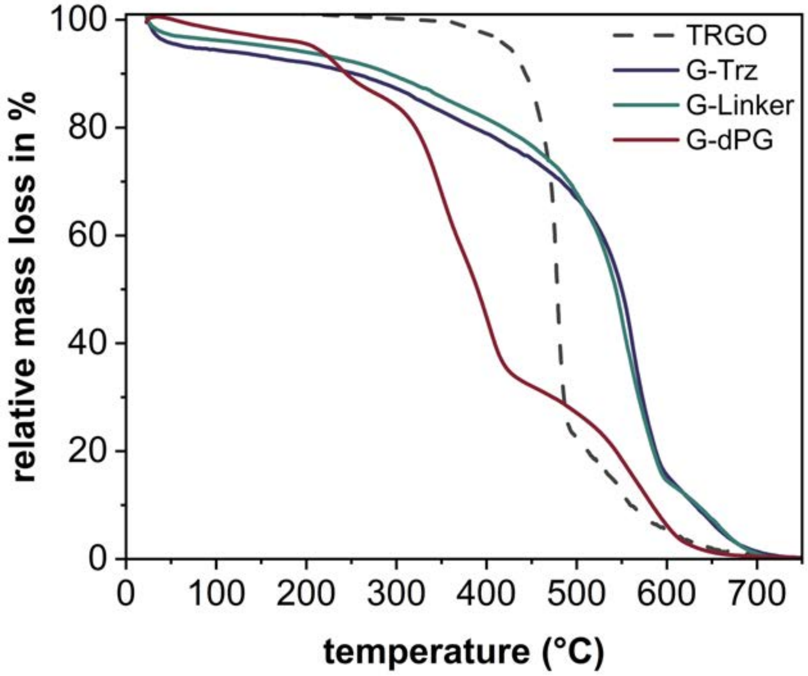

Figure S9. TGA thermograms of G, G-Trz, G-Linker and G-dPG.

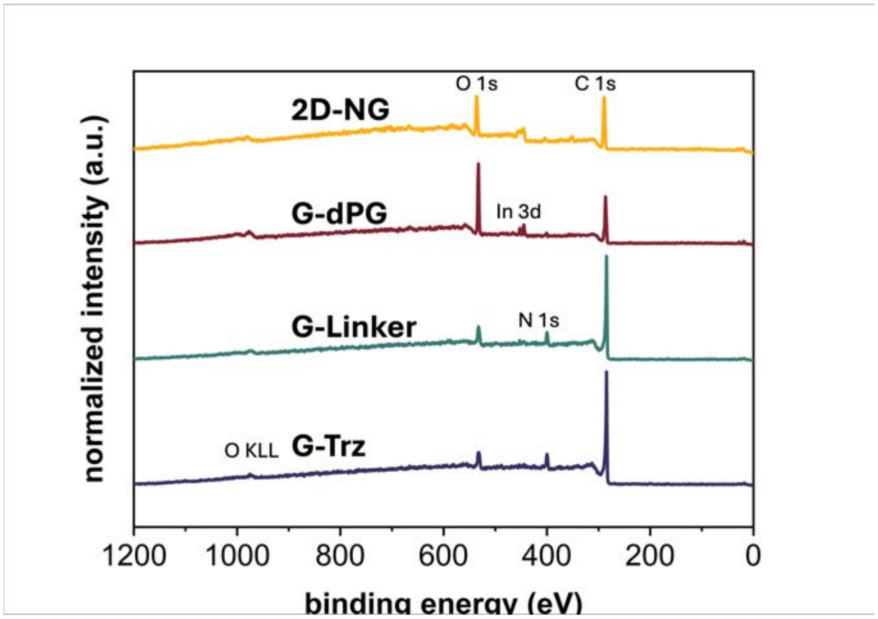

Figure S10. XP survey spectra of graphene intermediates.

Table S2. Relative element fractions and O/C atomic ratio obtained by XPS survey spectra.

Sample	O/C ratio	N/C ratio	C at%
G-Trz	0.09	0.11	83.2
G-Linker	0.10	0.07	87.0
G-dPG	0.45	0.05	66.2
2D-NG	0.32	0.05	69.7

Table S3. Fitting parameters of XPS spectra of G-Trz, G-Linker, G-dPG and 2D-NG.

Sample	Spectrum	Binding energy	L-G Mixing	FWHM	Interpretation	Abs. Area	Relat. Area
G-Trz	C1s	284.8	0.39	0.8	C-C/C=C	6851	0.76
		286.1	0.39	1.1	C-N	1381	0.15
		287.1	0.39	1.1	C-O, C-Cl	478	0.05
		288.4	0.39	1.1	C=O	311	0.03
		290.6	0.39	1.1	π-π* shake ups	173	0.01
	N1s	399.0	0.31	1.2	C-N-C	1479	0.40
		400.6	0.31	2.0	C-N=C/C-N=O	2244	0.60
G-Linker	C1s	284.8	0.39	0.9	C-C/C=C	10284	0.75
		286.5	0.39	1.2	C-N	2183	0.16
		287.2	0.39	1.2	C-O	845	0.06
		288.3	0.39	1.2	C=O	468	0.03
	N1s	398.9	0.31	2.0	C-N-C	1154	0.39
		400.5	0.31	0.9	C-N=C/C-N=O	1738	0.60
G-dPG	C1s	284.8	0.39	0.9	C-C/C=C	1033	0.18
		286.0	0.39	1.1	C-N	956	0.16
		287.1	0.39	1.3	C-O	3219	0.55
		289.8	0.39	1.3	C=O	655	0.11
	N1s	399.3	0.31	1.3	C-N-C	473	0.46
		401.3	0.31	1.7	C-N=C/C-N=O	557	0.54
2D-NG	C1s	284.8	0.39	1.4	C-C/C=C	1261	0.14
		285.8	0.39	1.4	C-O-C	2298	0.25
		286.9	0.39	1.4	C-O	3717	0.41
		288.3	0.39	1.4	O-C=O	1761	0.19

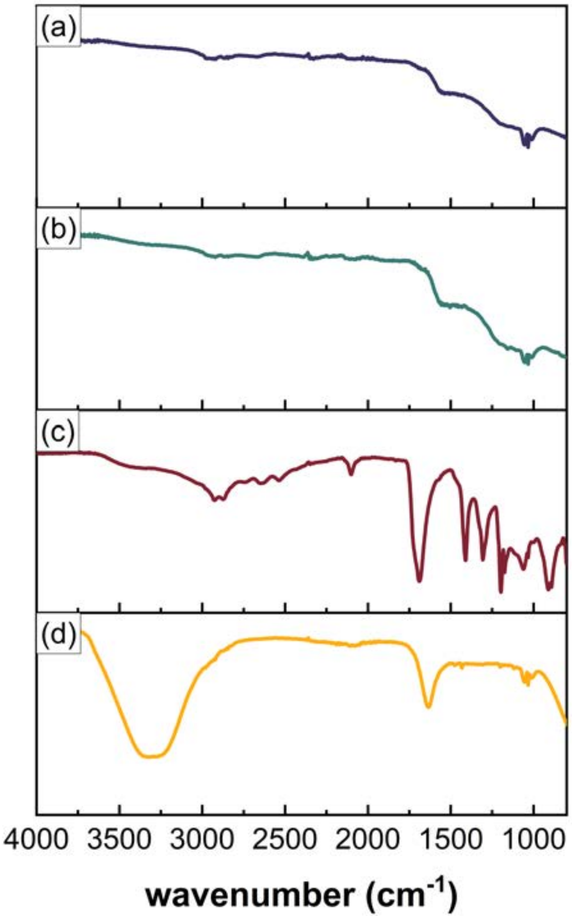

Figure S11. FTIR spectra of (a) G-Trz, (b) G-Linker, (c) G-dPG, and (d) 2D-NG.

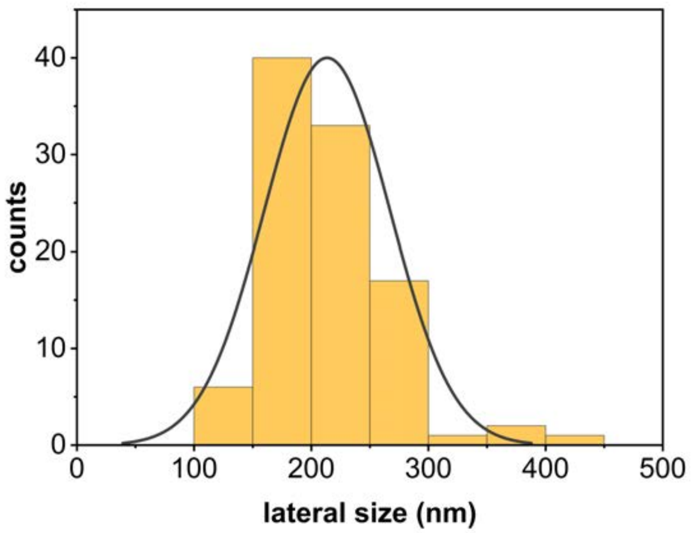

Figure S12. Lateral size distribution of 2D-NG, calculated by TEM (n = 100).

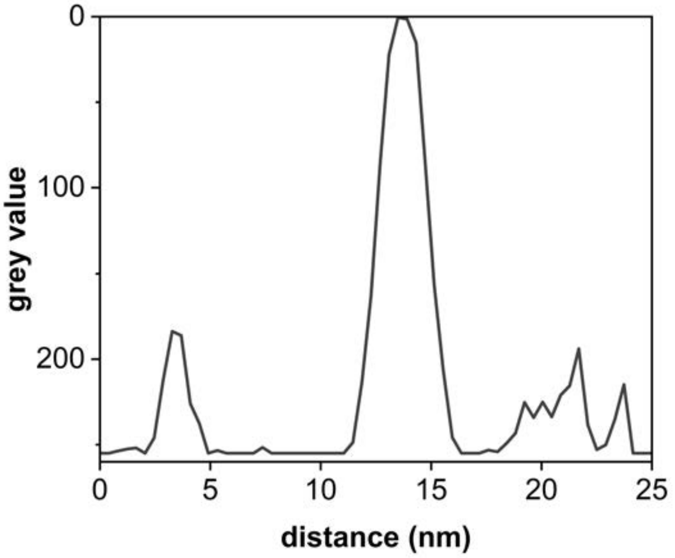

Figure S13. Thickness determination of 2D-NG through grey value analysis (n = 10).

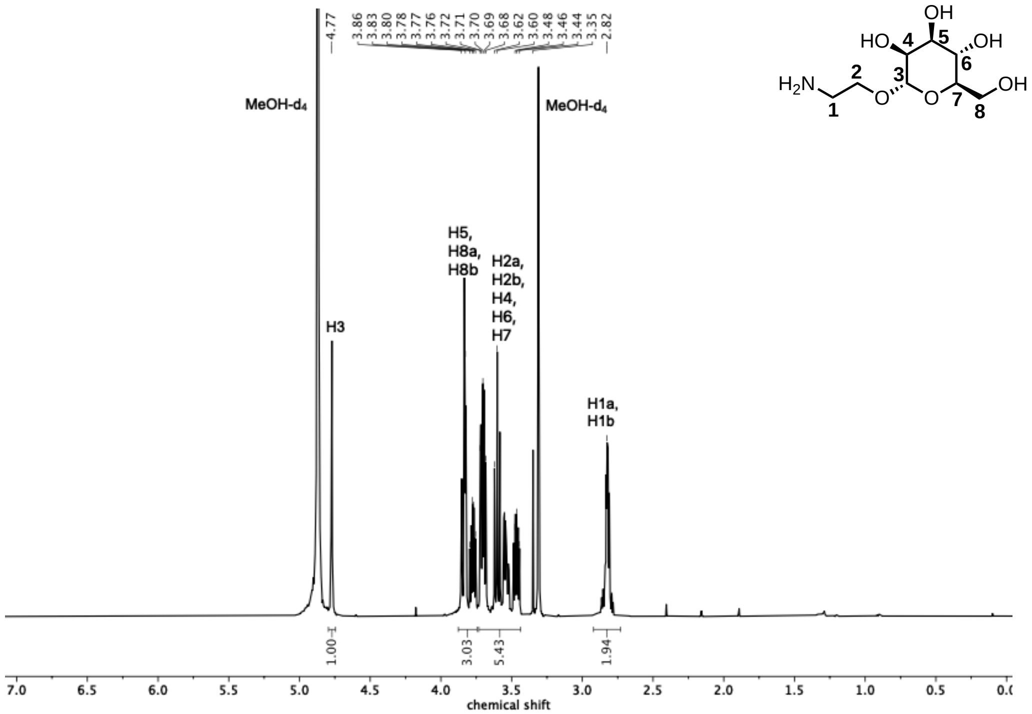

Figure S14. ^{1}H NMR spectrum of Man-NH_2 **14** recorded in MeOH-d_4.

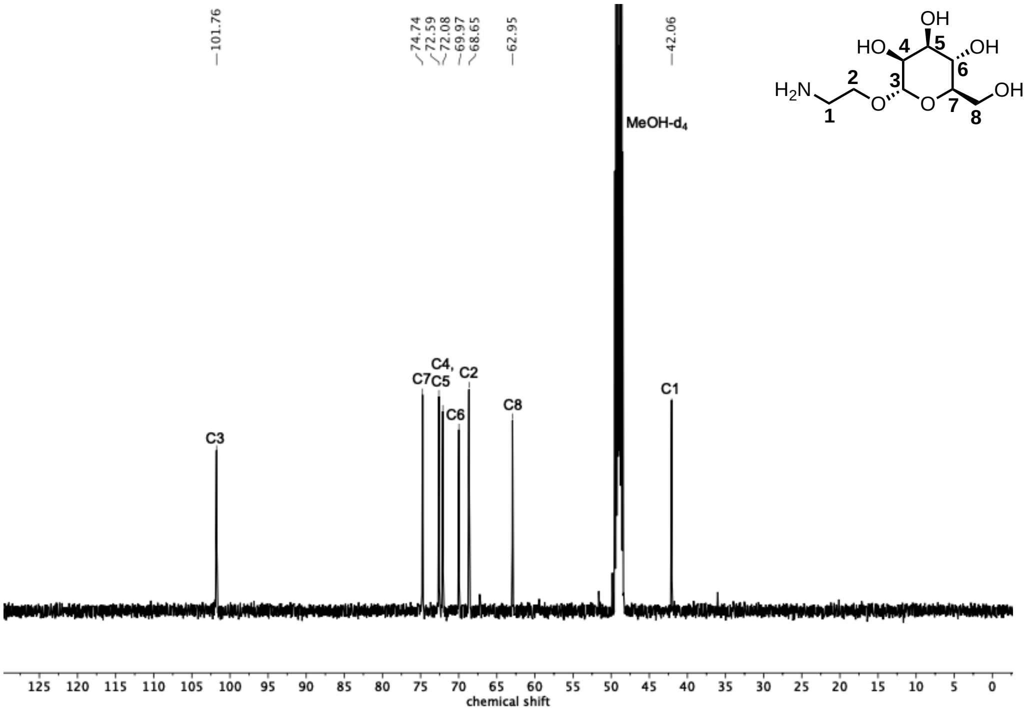

Figure S15. ^{13}C NMR spectrum of Man-NH_2 **14** recorded in MeOH-d_4.

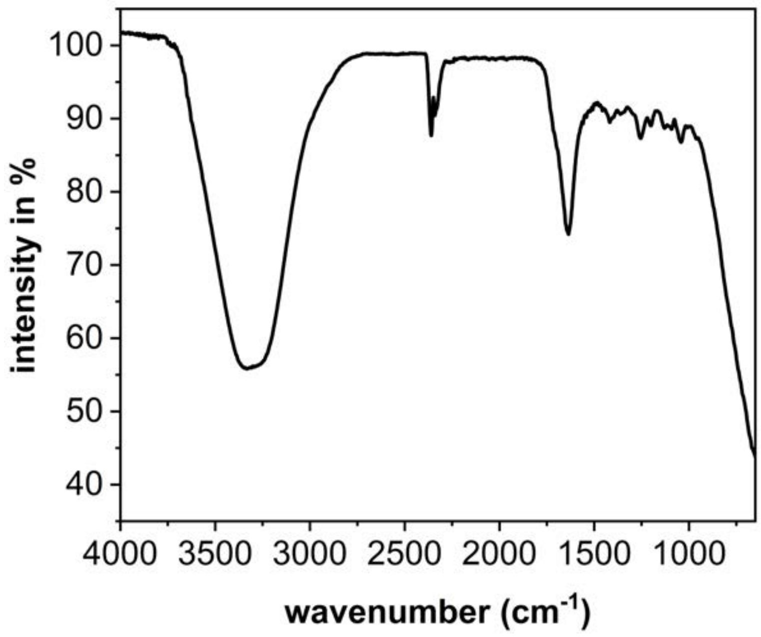

Figure S16. FTIR spectrum of Man-NH_2 **14**.

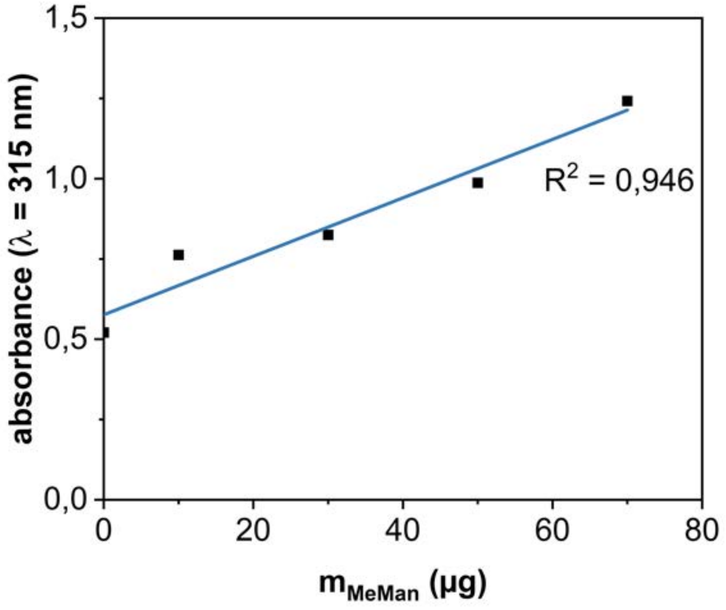

Figure S17. Calibration curve of MeMan for the H_2SO_4/UV-Vis assay.

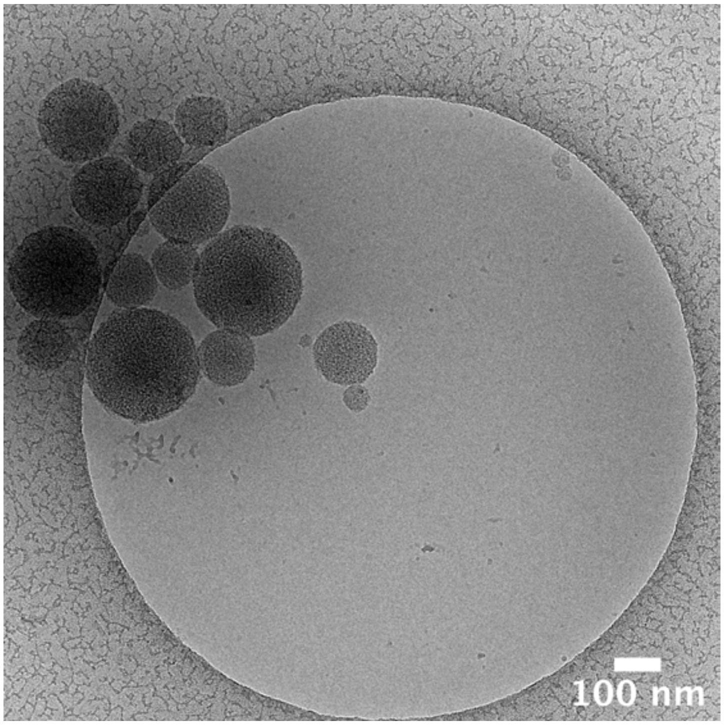

Figure S18. Cryo-TEM image of 3D-Man **8**.

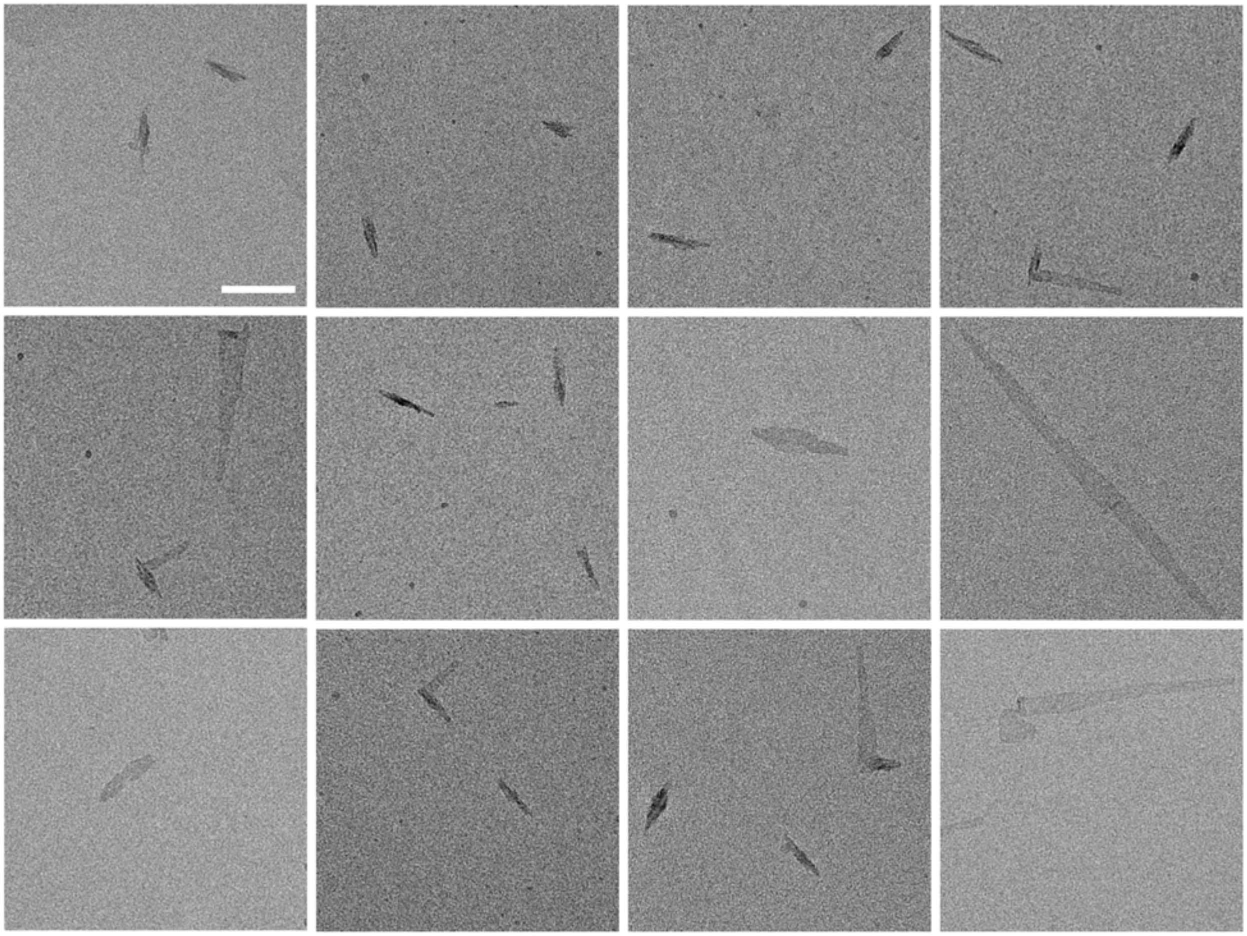

Figure S19. TEM images of 2D-Man **13** (scale bar is 100 nm).

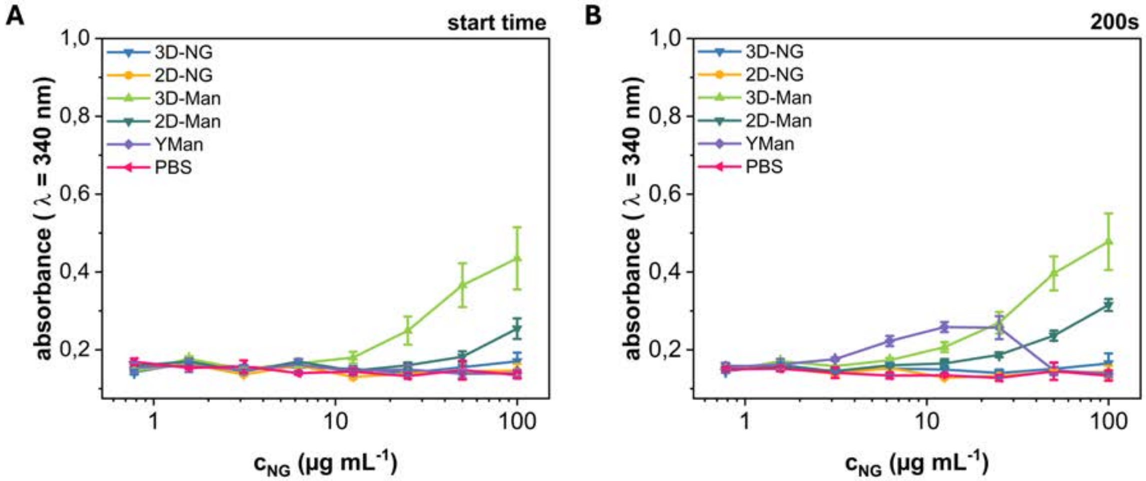

Figure S20. Aggregate formation by ConA (c = 150 µg mL^{-1}) and varying concentrations of NGs or yeast mannan (YMan), measured by turbidity. Time points (A) immediately after mixing of the two components of the assay and (B) 200 s afterwards.

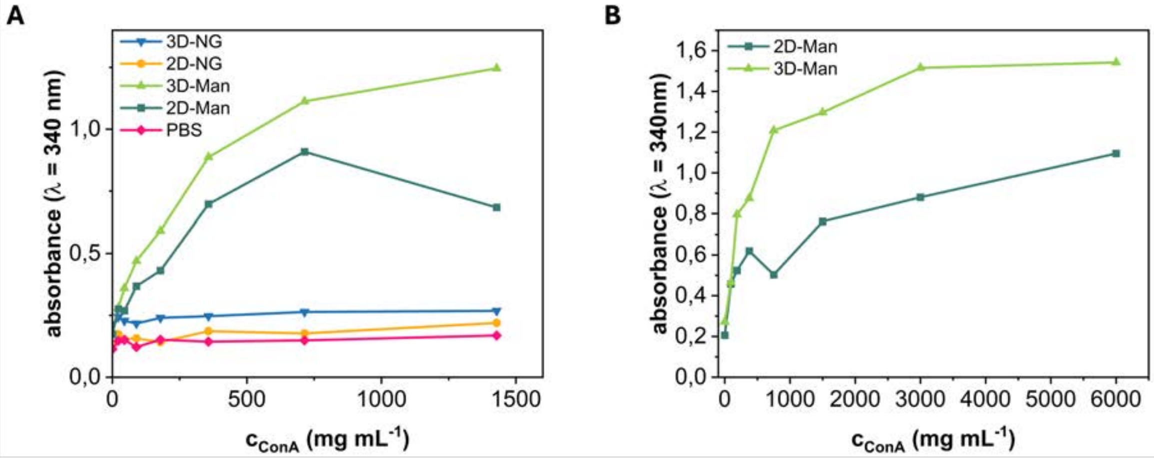

Figure S21. Precipitate formation by ConA (varying concentrations) and fixed concentration of NGs (c = 80 µg mL^{-1}) and controls, measured against turbidity. Plot of endpoint turbidity vs. concentration. (A) Concentration of ConA ranges from 0 to 1500 µg mL^{-1}. (B) Concentration of ConA ranges from 0 to 6000 µg mL^{-1}.

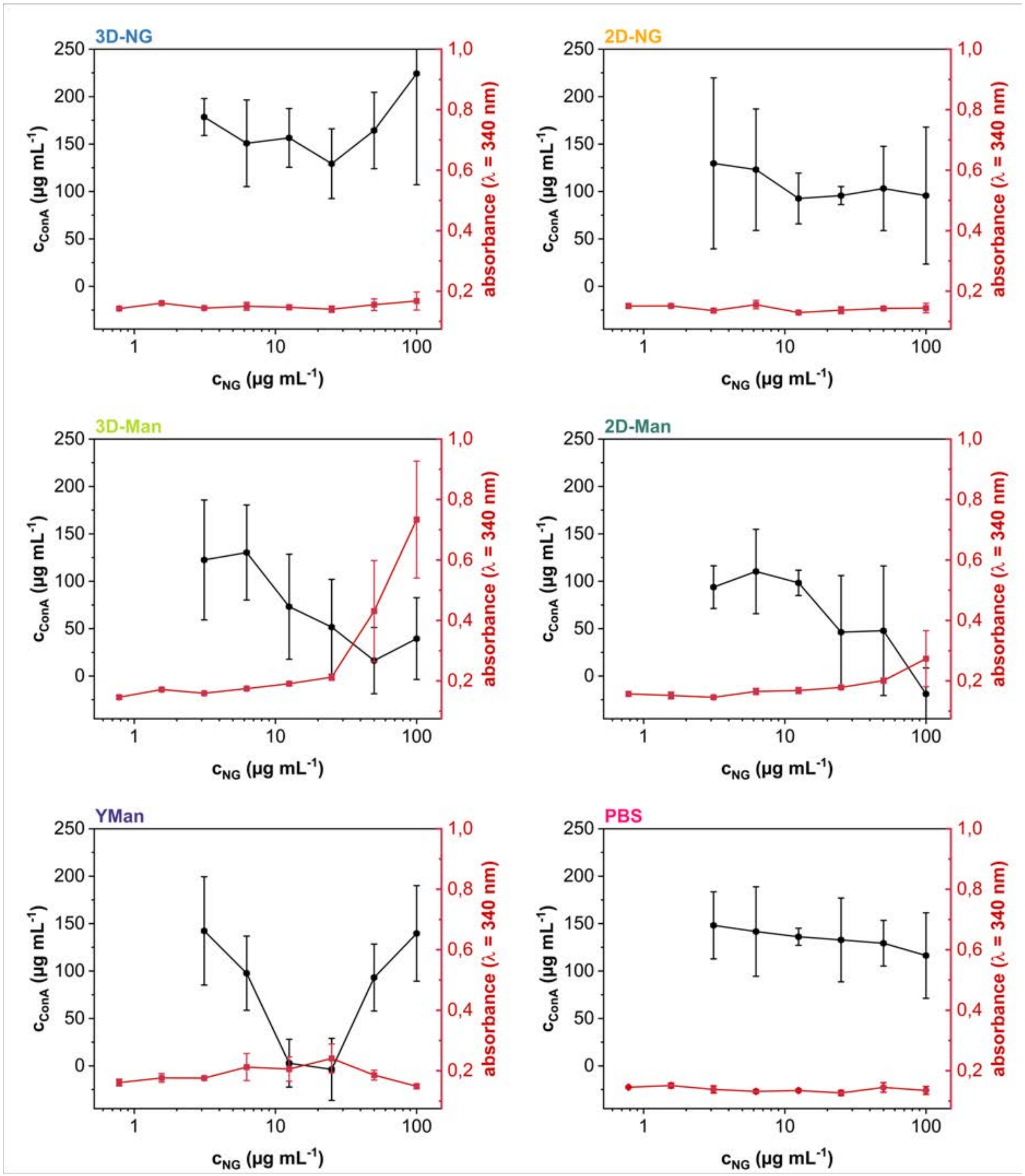

Figure S22. Comparison of ConA concentration from the supernatant of precipitate reaction wells as determined by Bradford assay with the turbidity changes of the NG samples and controls in dependence of NG concentration.

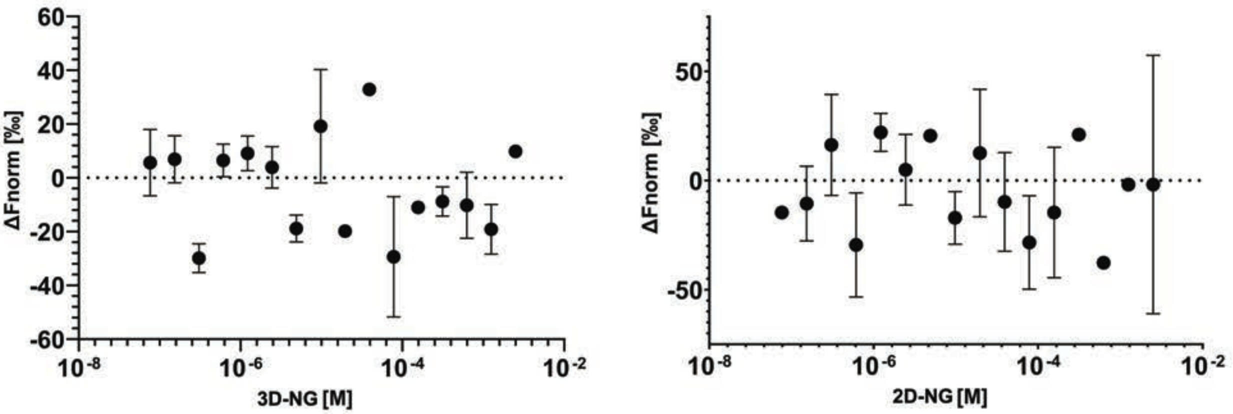

Figure S23. Binding analysis of non-mannosylated NGs (3D-NG and 2D-NG) to fluorescently labeled *E. coli* by microscale thermophoresis (MST) (n = 3).

1. S. Roller, H. Zhou and R. Haag, *Mol. Diversity*, 2005, **9**, 305-316.
2. A.-M. Faucher and C. Grand-Maître, *Synth. Communc.*, 2003, **33**, 3503-3511.
3. D. Steinhilber, T. Rossow, S. Wedepohl, F. Paulus, S. Seiffert and R. Haag, *Angew. Chem. Int. Ed.*, 2013, **52**, 13538-13543.
4. J. Dommerholt, S. Schmidt, R. Temming, L. J. Hendriks, F. P. Rutjes, J. C. van Hest, D. J. Lefeber, P. Friedl and F. L. van Delft, *Angew. Chem. Int. Ed.*, 2010, **49**, 9422-9425.
5. J. Ni, S. Singh and L.-W. Wang, *Bioconjug. Chem.*, 2003, **14**, 232-238.
